# Supplementary material for: What are the social predictors of accident and emergency attendance in disadvantaged neighbourhoods? Results from a cross-sectional household health survey in the north west of England
Source: BMJ Open. 2019 Jan 6;9(1):e022820. doi: 10.1136/bmjopen-2018-022820 (PMC6326270; doi:10.1136/bmjopen-2018-022820)
Supplement: Supplementary file 1 [file bmjopen-2018-022820supp001.pdf]

Appendix 1: Table of Measures

Variable	Source	Description/Example	Original scoring	Recoding for analysis
Age	Office for National Statistics	N/A	1 = Under 16 years<br>2 = 16-17 years<br>3 = 18-24 years<br>4 = 25-34 years<br>5 = 35-44 years<br>6 = 45-54 years<br>7 = 55-64 years<br>8 = 65-74 years<br>9 = 75+ years	1 = 18-24 years<br>2 = 25-44 years<br>3 = 45-64 years<br>4 = 65+ years
Sex	Office for National Statistics	N/A	1 = Male<br>2 = Female<br>3 = Other	1 = Male<br>2 = Female
Ethnicity	Office for National Statistics	N/A	1 = English / Welsh / Scottish / Northern Irish / British 2 = Irish<br>3 = Gypsy or Irish Traveller<br>4 = Any other White background, *please specify*<br>5 = White and Black Caribbean<br>6 = White and Black African<br>7 = White and Asian<br>8 = Any other Mixed / Multiple ethnic background, *please specify* 9 = Indian<br>10 = Pakistani<br>11 = Bangladeshi<br>12 = Chinese<br>13 = Any other Asian background, *please specify*<br>14 = African<br>15 = Caribbean<br>16 = Any other Black / African / Caribbean background, *please specify*<br>17 = Arab<br>95 = Any other group, please specify	0 = White<br>1 = BME
Non-	Office for National	N/A	1 = Going to school or college full time (including on vacation)	0 = employed

employment	Statistics		2 = In paid employment or self employed (or temporarily away)<br>3 = On a Government scheme for employment training<br>4 = Doing unpaid work for a business that you own, or that a relative owns<br>5 = Waiting to take up paid work already obtained<br>6 = Looking for paid work or a Government training scheme 7 = Intending to look for work but prevented by temporary sickness or injury<br>8 = Permanently unable to work because of long-term sickness or disability 9 = Retired from paid work<br>10 = Looking after the home or family<br>95 = Doing something else, specify	1 = not employed
Education	Office for National Statistics	Do you have any educational qualifications for which you received a certificate?<br><br>Do you have any professional, vocational or other workrelated qualifications for which you received a certificate?<br><br>What is your highest qualification?	1 = Yes, 2 = No<br><br>1 = Yes, 2 = No<br><br>1 = At degree level or above, 2 = Another kind of qualification	1 = No qualifications 2 = Professional or vocational qualification 3 = Degree or higher
Housing quality	English Housing Survey	During the winter months, does condensation form on the windows or walls of any room in your home apart from the bathrooms or toilets?<br><br>During the winter months, are there patches of mould or fungus in any room in your home, apart from bathrooms or toilets?	1 = Yes, 2 = No, 3 = Spontaneous: don’t know<br><br>1 = Yes, 2 = No, 3 = Spontaneous: don’t know	0 = No problems<br>1 = One problem<br>2 = Two problems 3 = Problems with all three issues

		During the cold winter weather, can you normally keep comfortably warm in your living room?	1 = Yes, 2 = No, 3 = Spontaneous: don't know	
Financial struggle	Wealth and Assets Survey	How well would you say your household is managing financially these days?	1 = Doing well<br>2 = Getting by<br>3 = Struggling	N/A
Financial Situation	Wealth and Assets Survey	Would you say your household is better off or worse off financially than you were a year ago?	1 = Better off<br>2 = About the same 3 = Worse off<br>4 = Don't know	1 = Better off<br>2 = About the same<br>3 = Worse off
Index of Multiple Deprivation (IMD)	Office of National Statistics	Measure of deprivation at level of Lower Level Super Output Area (LSOA) across seven domains: Income, Employment, Education, Health and Disability, Crime, Housing, ad Living Environment.	N/A	N/A
Physical Health	EuroQual Five Dimensional Scale (EQ-5D)	Standardised instrument that assesses problems with mobility, self-care, engagement in usual activities, and pain.	1 = No problems<br>2 = Some Problems/Moderate problems<br>3 = Extreme problems/Unable	0 = No problems 1 = Some/Severe problems
Multimorbidity	Psychiatric Morbidity Survey	Have you ever had any of [these health conditions] over the past 12 months?	1 = Cancer<br>2 = Diabetes<br>3 = Epilepsy/fits<br>4 = Migraine or other frequent headaches<br>5 = Dementia or Alzheimer's disease<br>6 = Any mental health issue<br>7 = Cataracts / eyesight problems (even if corrected with glasses or contacts)<br>8 = Ear/hearing problems (even if corrected with a hearing aid)<br>9 = Stroke	*At least one condition*<br><br>0 = No conditions<br>1 = One condition<br><br>*Multimorbidity*<br><br>0 = One condition 1 = More than one condition

			10 = Heart attack/angina<br>11 = High blood pressure<br>12 = Bronchitis/emphysema<br>13 = Asthma<br>14 = Allergies<br>15 = Stomach ulcer or other digestive problems<br>16 = Liver problems<br>17 = Bowel/colon problems<br>18 = Bladder problems/incontinences<br>19 = Arthritis<br>20 = Bone, back joint or muscle problems<br>21 = Gout<br>22 = Skin problems<br>95 = Other, please specify<br>96 = None of these	
Depression	Patient Health Questionnaire (PHQ-9)	Assesses how often participants had been bothered by pro problems such as "Feeling down, depressed, or hopeless" over the past two weeks.	1 = Not at all<br>2 = Several days<br>3 = More than half the days<br>4 = Nearly every day	N/A
Anxiety	Generalised Anxiety Disorder Questionnaire (GAD-7)	Assesses frequency of events such as "Being so restless that it is hard to sit still" over the past two weeks.	1 = Not at all<br>2 = Several days<br>3 = More than half the days<br>4 = Nearly every day	N/A
Paranoia	Five-item Persecution and Deservedness Scale (PaDS-5)	Assesses the extent to which people are suspicious of others' intentions. Participants rate their level of agreement with statements such as "I'm often suspicious of other people's intentions towards me."	1 = Strongly disagree<br>2 = Disagree<br>3 = Neither agree or disagree<br>4 = Agree<br>5 = Strongly agree	N/A
Social Support	Community Life Survey	Assesses the extent to which participants agree they receive *practical support* ("If I	1 = Definitely agree<br>2 = Tend to agree<br>3 = Tend to disagree	0 = Disagree<br>1 = Agree

		needed help, there are people who would be there for me") and *social contact* (If I wanted company or to socialise, there are people I can call on")	4 = Definitely disagree<br>5 = Don't know	
Healthcare Access	N/A	The *proximity to A&E departments* and *proximity to GP practices* was estimated using the Routino open source tool (Routino.org.uk) to calculate the shortest road distance in kilometres between the centre of each postcode and these health facilities. The average distance for all postcodes within each Lower layer Super Output Area (LSOA) level was then estimated and linked to survey responses based on the LSOA in which the respondent lived.	N/A	N/A
Healthcare usage	SANAD II trial	Have you been to a hospital casualty/A&E/ urgent care department in the past 12 months?	1=Yes; 2=No<br>If yes, please specify total number	N/A
