# Supplementary material for: What are the social predictors of accident and emergency attendance in disadvantaged neighbourhoods? Results from a cross-sectional household health survey in the north west of England
Source: BMJ Open. 2019 Jan 6;9(1):e022820. doi: 10.1136/bmjopen-2018-022820 (PMC6326270; doi:10.1136/bmjopen-2018-022820)
Supplement: Author's manuscript [file bmjopen-2018-022820.draft_revisions.pdf]

BMJ Open

BMJ Open is committed to open peer review. As part of this commitment we make the peer review history of every article we publish publicly available.

When an article is published we post the peer reviewers' comments and the authors' responses online. We also post the versions of the paper that were used during peer review. These are the versions that the peer review comments apply to.

The versions of the paper that follow are the versions that were submitted during the peer review process. They are not the versions of record or the final published versions. They should not be cited or distributed as the published version of this manuscript.

BMJ Open is an open access journal and the full, final, typeset and author-corrected version of record of the manuscript is available on our site with no access controls, subscription charges or pay-per-view fees (http://bmjopen.bmj.com).

If you have any questions on BMJ Open's open peer review process please email editorial.bmjopen@bmj.com

BMJ Open

What are the social predictors of A&E attendance in deprived neighbourhoods? Results from a cross-sectional household health survey in the North West of England

Journal:	*BMJ Open*
Manuscript ID	bmjopen-2018-022820
Article Type:	Research
Date Submitted by the Author:	07-Mar-2018
Complete List of Authors:	Giebel, Clarissa; University of Liverpool, Institute of Translational Medicine<br>McIntyre, Jason<br>Daras, Konstantinos<br>Gabbay, Mark<br>Downing, Jennifer; University of Liverpool, Department of Molecular and Clinical Pharmacology<br>Pirmohamed, Munir; The University of Liverpool, Department of Pharmacology<br>Walker, Fran<br>Sawicki, Woiciech<br>Alfirevic, Ana<br>Barr, Ben
Keywords:	accident and emergency, service access, Depression & mood disorders < PSYCHIATRY, deprivation, housing quality

SCHOLARONE™
Manuscripts

What are the social predictors of A&E attendance in deprived neighbourhoods? Results from a cross-sectional household health survey in the North West of England

Clarissa Giebel* *Research Manager*[1,2], Jason C. McIntyre* *Research Associate*[1,2], Konstantinos Daras *Data Scientist*[3], Mark Gabbay *Professor*[1,2], Jennifer Downing *Research Manager*[2,4], Munir Pirmohamed *Professor*[2,4], Fran Walker *Public Advisor*[2], Wojciech Sawicki *Consultant*[5], Ana Alfirevic* *Reader*[2,4], Ben Barr* *Senior Clinical Lecturer*[1,2]

[1]Institute of Psychology, Health and Society, University of Liverpool, England; [2]NIHR Collaboration for Leadership in Applied Health Research and Care North West Coast; [3]Geographic Data Science Lab, University of Liverpool, England; [4]Institute of Translational Medicine, University of Liverpool, England; [5]Royal Liverpool and Broadgreen University Hospitals NHS Trust, Liverpool, England

*joint first authors, and joint senior authors

Correspondence to: Clarissa Giebel, First Floor Waterhouse Building A Block, Institute of Translational Medicine, University of Liverpool, Liverpool, England. Email: Clarissa.giebel@liverpool.ac.uk; Tel.: +44 (0) 151 795 5390

For peer review only

Total word Count: 2,341
Keywords: Accident and emergency attendance; service access; depression; deprivation; housing quality

Abstract

Objectives The aim of this study was to identify the most important determinants of A&E attendance in deprived areas.

Design, setting, and participants A total of 3,510 residents from 20 deprived neighbourhoods in the North West Coast area in England completed a comprehensive public health survey.

Main outcome measures Participants were asked to complete general background information, as well as information about their physical health, mental health, lifestyle, social issues, housing and environment, work and finances, and health care service usage. Only one resident per household could take part in the survey. Poisson regression analysis was employed to assess the predictors of having attended A&E in the previous 12 months.

Results Being younger and having depression were both associated with higher levels of Accident and Emergency (A&E) attendance among residents from deprived neighbourhoods. Of the socio-economic factors, being unemployed and living in poor housing increased the likelihood of attending an A&E service. Service access was also found to be linked to attendance rates, with longer distance from a general practitioner (GP) service and shorter distance to A&E services increasing the likelihood of A&E attendance.

Conclusions This is one of the first surveys to explore a comprehensive set of socio-economic factors as well as proximity to both GP and A&E services as predictors of A&E attendance in deprived areas. Findings from this study suggest the need to address both socioeconomic issues, such as employment and housing quality, as well as structural issues, such as public transport, to reduce the current burden on A&E departments.

For peer review only - http://bmjopen.bmj.com/site/about/guidelines.xhtml

Strengths and limitations of this study

- This survey is novel insofar as it examines a comprehensive set of factors linked to A&E attendance, including physical health, mental health, service access and socio-economic factors.
- Participants were recruited from a wide geographical area of deprived neighbourhoods, which strengthens the representativeness of the survey findings.
- Households were approached at different times during the day to produce a less biased sample.
- Some of the most deprived people could not be captured in the survey due to not having a fixed address.

For peer review only

For peer review only - http://bmjopen.bmj.com/site/about/guidelines.xhtml

INTRODUCTION

Accident and emergency (A&E) attendance rates are rapidly increasing in the UK and are particularly high in deprived areas. This is leading to unsustainable pressure on the NHS, with 20.5 million attendances reported in England from April 2015 to March 2016, compared to 19.5 million the previous year [1]. According to a King's Fund report, unnecessary A&E attendances could be reduced by between eight and 18% if patients attended a primary care service for their health concerns instead [2]. The predictors of A&E attendance are complex and involve multiple components. Whilst we know that A&E attendances are high in deprived areas [3], the specific reasons for this are less clear. The prevalence of health problems is likely to be important; however, other factors may also play a role. Housing problems and employment status, for example, have been associated with high levels of A&E attendance, as have loneliness and low levels of social support [4,5]. Health service factors, including proximity to A&E and poor access to primary care, have also been linked to higher A&E attendance rates in past research [3, 6-11].

Previous studies on predictors of A&E attendance have been limited because they have not assessed the independent influence of the full range of potential risk factors, including morbidity, socio-economic factors, social support and proximity to A&E and primary care. In a recent study by Hull and colleagues [12], primary and secondary care data were linked with A&E attendance in London. The data showed that multimorbidity, smoking, increased number of GP attendances, shorter distance to A&E, and age were all linked to visits to an A&E department. By comparing the most deprived *versus* the least deprived quintile, Hull and colleagues [12] reported a 52% higher A&E attendance in the most deprived population sample.

The Household Health Survey

The Northwest Coast area of England contains some of the most deprived neighbourhoods in the country [13]. The Household Health Survey (HHS) of the National Institute for Health Research Collaboration for Leadership in Applied Health Research and Care in the North West Coast (NIHR

For peer review only - http://bmjopen.bmj.com/site/about/guidelines.xhtml

CLAHRC NWC) was conducted in 2015 to identify individual and neighbourhood level factors that contribute to inequalities in physical and mental health. Twenty deprived neighbourhoods were initially selected by participating local authorities. A random sample of households was then drawn from these neighbourhoods and one randomly selected respondent completed a face-to-face interview in each household (N=3,510). The survey combines information on a wide range of socioeconomic factors, mental and physical morbidities, and healthcare utilisation. Full details of the survey methodology are published elsewhere [14].

Aim

The aim of this study was to identify the most important determinants of A&E attendance in deprived neighbourhoods to identify pathways to reduce demand for A&E services.

METHODS

Participants

Participants were recruited from 20 deprived neighbourhoods across the Northwest Coast area of England. Neighbourhoods were selected and considered as deprived if they (1) had a population that was likely to be impacted by resilience interventions; (2) had a population between 5,000 and 10,000 residents; (3) had a deprivation index (Index of Multiple Deprivation) in the bottom 10% of the national average; (4) had a coherent or shared sense of identity among residents; and (5) had a local infrastructure to implement resilience initiatives. The Index of Multiple Deprivation (IMD) contains seven areas of deprivation, including income, employment, health deprivation and disability, education, skills, and training, barriers to housing and services, crime, as well as living environment. From each household, only one resident could participate, and the participant had to be 18 years or older. The 20 neighbourhoods comprised 10 deprived neighbourhoods in which a public health initiative was conducted after data collection, and 10 deprived comparator

For peer review only - http://bmjopen.bmj.com/site/about/guidelines.xhtml

neighbourhoods. The intended sample size for each of the first types of deprived neighbourhoods was 200, and 150 for those neighbourhoods without a public initiative.

Prior to the survey, ethical approval was obtained from the University of Liverpool (Ref: RETH000836). The survey was conducted by BMG research and interviews were completed between August 2015 and January 2016. A pilot survey was completed with 36 residents from the deprived neighbourhoods prior to the full data collection, which resulted in minor changes to survey documents.

Survey variables

The survey collected diverse information, including demographic details, employment status, housing quality, finances and social housing, as well as physical health, mental health, and healthcare service usage.

Public involvement

Members of the public have been involved throughout the design and implementation of the survey, in addition to external partners and representatives from local authorities. This involvement included attending research and design meetings, providing feedback on study documents, as well as helping with the dissemination of findings. Public advisors have been recruited from the sampled deprived areas and receive monetary reimbursement for their involvement.

Data analysis

Data were analysed using STATA version 14. Because the A&E attendance variable comprised highly skewed ($S\text{-}W = .39$, $p < .001$) count data, we constructed a Poisson regression model. Standard errors were adjusted to account for the multi-stage nature of the survey sampling, and the analysis was weighted for non-response. Demographic, socio-economic, health condition,

For peer review only - http://bmjopen.bmj.com/site/about/guidelines.xhtml

disability, symptom, and healthcare access variables were entered into the model as predictors of number of A&E attendances over the previous twelve months.

The proximity to A&E departments and GP practices was estimated using the Routino open source tool[1] to calculate the shortest road distance between the center of each postcode and these health facilities. The average distance for all postcodes within each Lower layer Super Output Area (LSOA) level[2] was then estimated and linked to survey responses based on the LSOA in which the respondent lived.

RESULTS

1,109 of the 3,510 survey respondents (31.6%) reported attending A&E at least once in the past 12 months. Among those, the number of attendances ranged from 1 to 95 (*m*=2.70, SD=4.90) and the mode was one attendance (n=561; 51%).

Table 1 shows the results of the Poisson regression model. Age was a significant demographic predictor of A&E attendances. 18-24 year olds were three times as likely to attend A&E compared to people over 64 years of age (RR=2.72, 95%CI 1.33-5.53). The health and disability variables predictive of A&E attendance included: having a health condition as opposed to no health condition (RR=1.78, 95%CI 1.19-2.66), having symptoms of depression (RR=1.36, 95%CI 1.01-1.83) and having problems with washing and dressing (RR=2.63, 95%CI 1.82-3.79). People with multiple morbidities were not more likely to attend A&E than people with only one condition. Of the socio-economic and environmental factors significantly associated with A&E attendance, being out of work increased risk of A&E attendance by 38% (RR=1.38, 95%CI 1.08-1.78) and living in a house with problems with cold, mould or damp increased risk of A&E attendance by 34% (RR=1.34, 95%CI 1.01 to 1.76). Surprisingly in this sample of people living in

[1] www.routino.org
[2] A Lower layer Super Output Area (LSOA) is a geographical area in the UK which is based on postcodes and clusters together groups of the population.

For peer review only - http://bmjopen.bmj.com/site/about/guidelines.xhtml

relatively deprived neighbourhoods, higher levels of education were associated with higher risk of A&E attendance. Levels of social support and social contact did not predict A&E attendance.

Both healthcare access variables were significantly related to A&E attendance. Specifically, living further from an A&E department (p<.001) reduced the likelihood of attending an A&E service by eight percent per kilometer, and living further from a GP (p<.05) increased the likelihood of attending A&E by 28 percent per kilometer, after controlling for health status, socioeconomic and demographic factors.

DISCUSSION

This is one of the first surveys to collect data on socio-economic background, mental and physical health and health care utilisation, and to combine these factors with distance to health care services. Spanning a wide geographical area in one of the most deprived areas of England [13], findings from the survey identify several key risk factors of A&E attendance rates, including young age, depression, high education, non-employment, poor housing, as well as longer distance from a GP and shorter distance to an A&E service.

Considering the increase in A&E attendance rates across England [1], particularly in deprived areas [3], it is important to understand the reasons behind this rise and to design strategies to reduce health inequalities. A&E attendance was significantly higher for 18 to 24 year olds, which has been found in previous work [15], and may be due to heightened rates of accidents, trauma and alcohol-related attendances at A&E [16], or higher use of A&E for primary care reasons in this age group. A limitation of our study is that we did not collect any information on the reasons for A&E attendance. Furthermore, A&E attendance has to be distinguished from admission to hospital, which we know is more prevalent in the elderly with long-term conditions [17].

Higher levels of education were linked to increased risks of attending A&E services in our deprived sample. Considering that people from a low socioeconomic background are more likely to

For peer review only - http://bmjopen.bmj.com/site/about/guidelines.xhtml

attend A&E services, as shown in this survey and supported by other research [18], it is perhaps surprising to find those with a professional or degree level education to be up to twice as likely to access A&E. One possible reason for this is that higher educated residents are more likely to recognise specific symptoms and become pro-active by seeking medical help. It is also plausible that people with higher qualifications may not expect to live in a deprived neighbourhood, which may impact on their mental well-being and lead to increased A&E attendances. Indeed, depression as well as problems with self-care were independently associated with more A&E visits, supporting previous evidence of increased emergency attendances among people with mental health problems who are more likely to be "frequent users" [6,19-20]. Thus, interventions that address mental health problems, particularly depression, and that provide greater support to people who have problems with self-care may be linked to concomitant declines in A&E attendance.

Being out of work and living in poor housing were important predictors of increased A&E attendance, above and beyond the effect of health status. This could be because these factors exacerbate existing conditions. Indeed, unemployment has been linked to higher A&E attendances for asthma [21], whilst poor housing quality has been linked to more asthma-related symptoms [22] and worse mental health [23]. Controlling for these socioeconomic factors the Index of Multiple Deprivation (IMD) was not independently associated with A&E attendance within this sample of highly deprived areas. This indicates that much of the high level of A&E attendance found by others in deprived areas [3] is probably explained by differences in the prevalence of health conditions, high unemployment and poor housing. Interventions that improve housing conditions and promote employment in deprived communities should be integral to strategies to reduce demand for A&E services.

We found that longer distance from GP practices and shorter distance to A&E departments were significant predictors of A&E attendance. The findings are consistent with previous work on A&E proximity [8,9;12]; however, we additionally identified GP practice proximity as a unique determinant of A&E attendance. The results indicate that primary care access predicts A&E

For peer review only - http://bmjopen.bmj.com/site/about/guidelines.xhtml

attendance over and above the effects of health status, socioeconomic status, and A&E access. The current trend of consolidating GP practices in fewer, larger health centres may therefore be contributing to increases in A&E attendances. This trend may also be linked to ease of access, in that patients may be unable to get a GP appointment and therefore attend an A&E service instead. Future approaches should be cognisant of primary care service placement and access. Reduced demand for A&E may be achieved via improved public transport links that consider primary care locations, or via strategically positioning primary care services in areas with high A&E attendance rates.

One recently conducted retrospective study has also explored the socioeconomic predictors of A&E attendance [12], whilst differing in several aspects from the household health survey. On the one hand, the study by Hull and colleagues [12] did not measure the effects of proximity to GP services, and calculated distance in actual distance as opposed our assessment of time distance, and the shortest time it takes to access a healthcare service. In addition, the household health survey contains a rich set of mental health and socioeconomic variables, whereas the London-based assessment of primary and secondary care data only employs the IMD as a measure of social deprivation, but no information on for example housing quality.

The findings from this survey should be considered in light of a few limitations. The cross-sectional nature of the survey only allowed investigation of the predictors of A&E attendance at one point in time. Moreover, this survey was based on self-reports, and thus participants had to remember whether they had attended an A&E department, and how often, in the past 12 months. This could be avoided by referring to Hospital Episodes Statistics data from the National Health Service. Lastly, the survey purposefully recruited residents from some of the most deprived areas within England and the Northwest Coast area. However, people without a fixed address who may be living in extremely deprived circumstances were not captured by our data.

For peer review only - http://bmjopen.bmj.com/site/about/guidelines.xhtml

Conclusions

We conducted a cross-sectional analysis of A&E attendance risk factors in a large sample of UK residents in deprived neighbourhoods. To our knowledge, this is the most comprehensive examination of diverse socioeconomic determinants of A&E attendance, and the first to examine GP proximity alongside A&E proximity. Housing quality, employment status, self-care difficulties, depression, and proximity to both primary and emergency services were identified as the most important determinants of A&E attendance. The findings highlight the need for multi-pronged approaches to reduce A&E attendances that address socioeconomic inequalities, such as employment and housing quality, but also structural issues such as access to primary care. Key policy suggestions likely to reduce A&E attendance based on these findings include: strategic placement of — and better transport links to — primary care services, better housing conditions, increased employment and improved support for self-care and public mental health. Strategies to reduce strain on health services need to address the social and economic factors that underpin demand for healthcare and enhance access to primary care.

For peer review only - http://bmjopen.bmj.com/site/about/guidelines.xhtml

Funding

This work was supported by the National Institute for Health Research, Collaboration for Leadership and Health Research and Care North West Coast (NIHR CLAHRC NWC). The views expressed are those of the author(s) and not necessarily those of the NHS, the NIHR or the Department of Health.

Competing interests

We have read and understood BMJ policy on declaration of interests and declare the following: All authors have completed the ICMJE uniform disclosure form. The authors have no financial relationships with any organisations that might have an interest in the submitted work in the previous three years, no other relationships or activities that could appear to have influenced the submitted work.

Author Contributions

CG and JMI jointly wrote the article, and JMI and BB performed statistical analysis. KD, MP, MG, JD, FW, WS, AA, and BB provided feedback on drafts of the manuscript and approved the final version.

Research ethics

The research was approved by the University of Liverpool Committee on Research Ethics (Ref: RETH00836). Participants provided informed consent before completing the study.

Data sharing statement

The dataset from the North West Coast Household Health Survey will be made publicly available after an embargo period.

For peer review only - http://bmjopen.bmj.com/site/about/guidelines.xhtml

References

[1] NHS Digital, 2017. Hospital Accident and Emergency Activity 2015-2016. Health and Social Care Information Centre, London: NHS Digital.

[2] Tian Y. 2012. Data briefing: emergency hospital admissions for ambulatory care-sensitive conditions. King's Fund report. https://www.kingsfund.org.uk/sites/default/files/field/field_publication_file/data-briefing-emergency-hospital-admissions-for-ambulatory-care-sensitive-conditions-apr-2012.pdf (accessed 28 Sep 2017).

[3] Scantlebury R, Rowlands G, Durbaba S, Schofield P, Sidhu K, Ashworth M. Socioeconomic deprivation and accident and emergency attendances: cross-sectional analysis of general practice in England. Br J Gen Pract 2015;65(639):e649-e654.

[4] Geller J, Janson P, McGovern E, Valdini A. Loneliness as a Predictor of Hospital Emergency Department Use. The Journal of Family Practice 1999;48(10): 801–4.

[5] Molloy GJ, McGee HM, O'Neill D, Conroy RM. Loneliness and Emergency and Planned Hospitalizations in a Community Sample of Older Adults. *Journal of the American Geriatrics Society* 2010;58(8): 1538–41.

[6] Daniels J, Osborn M, Davis C. Better safe than sorry? Frequent attendance in a hospital emergency department: an exploratory study. Br J Pain 2017; doi:10.1177/2049463717720635

[7] Hull SA, Jones IR, Moser K. Factors influencing the attendance rate at accident and emergency departments in East London: The contributions of practice organization, population characteristics and distance. J Health Services Research Policy 1997;2(1):6-13.

[8] Paul P, Heng BH, Seow E, Molina J, Tay SY. Predictors of frequent attenders of emergency department at an acute general hospital in Singapore. Emergency Medical Journal 2010;27:805-805.

[9] Rudge GM, Mohammed MA, Fillingham SC, et al. The Combined Influence of Distance and Neighbourhood Deprivation on Emergency Department Attendance in a Large English Population: A Retrospective Database Study. PLOS One 2013; doi: 10.1371/journal.pone.0067943

[10] Takahashi PY, Ryu E, Hathcock MA, et al. A novel housing-based socioeconomic measure predicts hospitalization and multiple chronic conditions in a community population. J Epidemiology Community Health 2016;70(3):286-291.

[11] Tammes P, Morris RW, Brangan E, et al. Exploring the relationship between general practice characteristics, and attendance at walk-in centres, minor injuries units and EDs in England 2012/2013: a cross-sectional study. BMJ 2017;33:702-708.

[12] Hull SA, Homer K, Boomla K, Robson J, Ashworth M. Population and patient factors affecting emergency department attendance in London: retrospective cohort analysis of linked primary and secondary care records. Br J Gen Pract 2018; doi: 10.3399/bjgp18X694397

[13] Department for Communities and Local Government. The English Indices of Deprivation 2015. Office of National Statistics, London: 2015.

[14] McIntyre J, Wickham S, Barr B, Bentall RP. Social Identity and Psychosis: Associations and Psychological Mechanisms. Schizophrenia Bulletin 2017, doi: 10.1093/schbul.sbx110.

[15] Health and Social Care Information Centre. *Hospital Accident and Emergency Activity: 2015-16*. NHS Digital, England: 2017.

For peer review only - http://bmjopen.bmj.com/site/about/guidelines.xhtml

[16] Parkinson K, Newbury-Birch D, Phillipson A, et al. Prevalence of alcohol related attendance at an inner city emergency department and its impact: a dual prospective and retrospective cohort study. Emergency Medicine Journal 2016;33:187-193.

[17] King's Fund. *Long-term conditions and multi-morbidity*. Last accessed 15th January 2018: https://www.kingsfund.org.uk/projects/time-think-differently/trends-disease-and-disability-long-term-conditions-multi-morbidity

[18] Krieg C, Hudon C, Chouinard MC, Dufour I. Individual predictors of frequent emergency department use: a scoping review. BMC Health Serv Res 2016; 16:594.

[19] Keene J, Rodriguez J. Are mental health problems associated with use of Accident and Emergency and health-related harm? European J Public Health 2007;17(4):387-393.

[20] Ingram WM, Weston C, Ritchie MD, Larson S. Depression linked to frequent emergency department use in large 10-year retrospective analysis of an integrated health care system. bioRxiv 2017; doi:10.1101/115238

[21] Forbes L, Harvey S, Newson R, et al. Risk Factors for Accident and Emergency (A&E) Attendance for Asthma in Inner City Children. Thorax 2007;62(10):855–60.

[22] Barton A, Basham M, Foy C, Buckingham K, Somerville M. The Watcombe Housing Study: the short term effect of improving housing conditions on the health of residents. Journal of Epidemiology Community Health 2007;61(9):771-777.

[23] Evans GW, Wells NM, Chan HYE, Saltzman H. Housing quality and mental health. J Consulting Clin Psychol 2000;68(3):526-530.

For peer review only

For peer review only - http://bmjopen.bmj.com/site/about/guidelines.xhtml

Socioeconomic factors	Odds ratio of A&E attendance	95% CI

Table 1. Determinants of number of A&E attendances over the previous 12 months (n=3,510)

For peer review only - http://bmjopen.bmj.com/site/about/guidelines.xhtml
For peer review only

**Demographics**		
Age (65+)		
18-24	2.72**	1.33, 5.53
25-44	1.43	.95, 2.16
45-64	1.21	.83, 1.77
Gender (female)	.90	.69, 1.18
Ethnicity (BME)	.60	.35, 1.04
**Socioeconomic status**		
Education (No qualifications)		
Professional, vocational or work-related Certificate	1.58**	1.17, 2.14
Degree or higher	2.00*	1.15, 3.48
Non-employment (Not in paid or self-employment)	1.38*	1.08, 1.78
Index of Multiple Deprivation	.99	.99, 1.00
Financial struggle (“Doing well”)		
“Getting by”	1.00	.72, 1.40
“Struggling”	1.22	.75, 1.98
Financial situation worse than 12 months ago	.99	.78, 1.26
**Housing quality**		
Problems with condensation, mold, and temperature	1.34*	1.01, 1.76
**Health status**		
Mobility problems (EQ5D)	1.24	.84, 1.83
Self-care problems (EQ5D)	2.63***	1.82, 3.79
Problems engaging in usual activities (EQ5D)	1.43*	1.01, 2.03
Pain (EQ5D)	1.24	.90, 1.71
1 condition (base category: no condition)	1.78**	1.19, 2.66
>1 condition (base category: 1 condition)	1.02	.67, 1.55
**Mental Health**		
Depression (PHQ-9)	1.36*	1.01, 1.83
Anxiety (GAD-7)	.86	.65, 1.14
Paranoia (PaDS-5)	1.04	.89, 1.21
**Social support**		
Practical support	.93	.47, 1.85
Social contact	1.08	.70, 1.70
**Healthcare access**		
Distance to GP	1.46**	1.12, 1.90
Distance to A&E	.93***	.89, .97

* $p < .05$, ** $p < .01$, *** $p < .001$

BME= Black and Minority Ethnic; EQ5D= Euroqol 5 dimensions; a measure of generic health status; GAD-7=Generalised Anxiety Disorder Assessment; PHQ-9=Patient Health Questionnaire; PaDS-5= Persecution and Deservedness Scale

For peer review only

For peer review only - http://bmjopen.bmj.com/site/about/guidelines.xhtml

STROBE Statement—checklist of items that should be included in reports of observational studies

	Item No	Recommendation	Page in manuscript
**Title and abstract**	1	(*a*) Indicate the study's design with a commonly used term in the title or the abstract	
		(*b*) Provide in the abstract an informative and balanced summary of what was done and what was found	Abstract
**Introduction**			
Background/rationale	2	Explain the scientific background and rationale for the investigation being reported	3
Objectives	3	State specific objectives, including any prespecified hypotheses	4
**Methods**			
Study design	4	Present key elements of study design early in the paper	4
Setting	5	Describe the setting, locations, and relevant dates, including periods of recruitment, exposure, follow-up, and data collection	4
Participants	6	(*a*) *Cohort study*—Give the eligibility criteria, and the sources and methods of selection of participants. Describe methods of follow-up<br>*Case-control study*—Give the eligibility criteria, and the sources and methods of case ascertainment and control selection. Give the rationale for the choice of cases and controls<br>*Cross-sectional study*—Give the eligibility criteria, and the sources and methods of selection of participants	4
		(*b*) *Cohort study*—For matched studies, give matching criteria and number of exposed and unexposed<br>*Case-control study*—For matched studies, give matching criteria and the number of controls per case	
Variables	7	Clearly define all outcomes, exposures, predictors, potential confounders, and effect modifiers. Give diagnostic criteria, if applicable	5
Data sources/ measurement	8*	For each variable of interest, give sources of data and details of methods of assessment (measurement). Describe comparability of assessment methods if there is more than one group	*5*
Bias	9	Describe any efforts to address potential sources of bias	
Study size	10	Explain how the study size was arrived at	5
Quantitative variables	11	Explain how quantitative variables were handled in the analyses. If applicable, describe which groupings were chosen and why	5
Statistical methods	12	(*a*) Describe all statistical methods, including those used to control for confounding	5
		(*b*) Describe any methods used to examine subgroups and interactions	
		(*c*) Explain how missing data were addressed	
		(*d*) *Cohort study*—If applicable, explain how loss to follow-up was addressed<br>*Case-control study*—If applicable, explain how matching of cases and controls was addressed<br>*Cross-sectional study*—If applicable, describe analytical methods taking account of sampling strategy	

For peer review only

(*e*) Describe any sensitivity analyses

Continued on next page

For peer review only

Results			Page in the manuscript
Participants	13*	(a) Report numbers of individuals at each stage of study—eg numbers potentially eligible, examined for eligibility, confirmed eligible, included in the study, completing follow-up, and analysed	6
		(b) Give reasons for non-participation at each stage	
		(c) Consider use of a flow diagram	
Descriptive data	14*	(a) Give characteristics of study participants (eg demographic, clinical, social) and information on exposures and potential confounders	6
		(b) Indicate number of participants with missing data for each variable of interest	
		(c) *Cohort study*—Summarise follow-up time (eg, average and total amount)	
Outcome data	15*	*Cohort study*—Report numbers of outcome events or summary measures over time	
		Case-control study—Report numbers in each exposure category, or summary measures of exposure	
		Cross-sectional study—Report numbers of outcome events or summary measures	*6*
Main results	16	(*a*) Give unadjusted estimates and, if applicable, confounder-adjusted estimates and their precision (eg, 95% confidence interval). Make clear which confounders were adjusted for and why they were included	6
		(*b*) Report category boundaries when continuous variables were categorized	
		(*c*) If relevant, consider translating estimates of relative risk into absolute risk for a meaningful time period	
Other analyses	17	Report other analyses done—eg analyses of subgroups and interactions, and sensitivity analyses	
**Discussion**			
Key results	18	Summarise key results with reference to study objectives	7-9
Limitations	19	Discuss limitations of the study, taking into account sources of potential bias or imprecision. Discuss both direction and magnitude of any potential bias	9
Interpretation	20	Give a cautious overall interpretation of results considering objectives, limitations, multiplicity of analyses, results from similar studies, and other relevant evidence	7-9
Generalisability	21	Discuss the generalisability (external validity) of the study results	7-10
**Other information**			
Funding	22	Give the source of funding and the role of the funders for the present study and, if applicable, for the original study on which the present article is based	11

*Give information separately for cases and controls in case-control studies and, if applicable, for exposed and unexposed groups in cohort and cross-sectional studies.

For peer review only

For peer review only - http://bmjopen.bmj.com/site/about/guidelines.xhtml

Note: An Explanation and Elaboration article discusses each checklist item and gives methodological background and published examples of transparent reporting. The STROBE checklist is best used in conjunction with this article (freely available on the Web sites of PLoS Medicine at http://www.plosmedicine.org/, Annals of Internal Medicine at http://www.annals.org/, and Epidemiology at http://www.epidem.com/). Information on the STROBE Initiative is available at www.strobe-statement.org.

For peer review only

For peer review only - http://bmjopen.bmj.com/site/about/guidelines.xhtml

BMJ Open

What are the social predictors of Accident & Emergency attendance in disadvantaged neighbourhoods? Results from a cross-sectional household health survey in the North West of England

Journal:	*BMJ Open*
Manuscript ID	bmjopen-2018-022820.R1
Article Type:	Research
Date Submitted by the Author:	11-Jun-2018
Complete List of Authors:	Giebel, Clarissa; University of Liverpool, Institute of Translational Medicine<br>McIntyre, Jason<br>Daras, Konstantinos<br>Gabbay, Mark<br>Downing, Jennifer; University of Liverpool, Department of Molecular and Clinical Pharmacology<br>Pirmohamed, Munir; The University of Liverpool, Department of Pharmacology<br>Walker, Fran<br>Sawicki, Wojciech<br>Alfirevic, Ana<br>Barr, Ben
**Primary Subject Heading**:	Public health
Secondary Subject Heading:	Health services research
Keywords:	accident and emergency, service access, Depression & mood disorders < PSYCHIATRY, deprivation, housing quality

SCHOLARONE™ Manuscripts

What are the social predictors of Accident & Emergency attendance in disadvantaged neighbourhoods? Results from a cross-sectional household health survey in the North West of England

Clarissa Giebel* *Research Manager*[1,2], Jason C. McIntyre* *Research Associate*[1,2], Konstantinos Daras *Data Scientist*[3], Mark Gabbay *Professor*[1,2], Jennifer Downing *Research Manager*[2,4], Munir Pirmohamed *Professor*[2,4], Fran Walker *Public Advisor*[2], Wojciech Sawicki *Consultant*[5], Ana Alfirevic* *Reader*[2,4], Ben Barr* *Senior Clinical Lecturer*[1,2]

[1]Institute of Psychology, Health and Society, University of Liverpool, England; [2]NIHR Collaboration for Leadership in Applied Health Research and Care North West Coast; [3]Geographic Data Science Lab, University of Liverpool, England; [4]Institute of Translational Medicine, University of Liverpool, England; [5]Royal Liverpool and Broadgreen University Hospitals NHS Trust, Liverpool, England
*joint first authors, and joint senior authors

Correspondence to: Clarissa Giebel, First Floor Waterhouse Building A Block, Institute of Translational Medicine, University of Liverpool, Liverpool, England. Email: Clarissa.giebel@liverpool.ac.uk; Tel.: +44 (0) 151 795 5390

For peer review only

Total word Count: 2,341
Keywords: Accident and emergency attendance; service access; depression; deprivation; housing quality

Abstract

Objectives The aim of this study was to identify the most important determinants of Accident & Emergency (A&E) attendance in disadvantaged areas.

Design, setting, and participants A total of 3,510 residents from 20 disadvantaged neighbourhoods in the North West Coast area in England completed a comprehensive public health survey.

Main outcome measures Participants were asked to complete general background information, as well as information about their physical health, mental health, lifestyle, social issues, housing and environment, work and finances, and health care service usage. Only one resident per household could take part in the survey. Poisson regression analysis was employed to assess the predictors of having attended A&E in the previous 12 months.

Results 31.6 percent of the sample reported having attended A&E in the previous 12 months, ranging from 1 to 95 visits. Controlling for demographic and health factors, not being in employment and living in poor quality housing increased the likelihood of attending an A&E service. Service access was also found to be predictive of attendance rates insofar as there was an additional 18 fewer A&E attendances per 100 population for each kilometre closer a person lived to a General Practitioner (GP) practice, and 3 fewer attendances per 100 population for each kilometre further a person lived from an A&E department.

Conclusions This is one of the first surveys to explore a comprehensive set of socio-economic factors as well as proximity to both GP and A&E services as predictors of A&E attendance in disadvantaged areas. Findings from this study suggest the need to address

For peer review only - http://bmjopen.bmj.com/site/about/guidelines.xhtml

both socioeconomic issues, such as employment and housing quality, as well as structural issues, such as public transport and access to primary care, to reduce the current burden on A&E departments.

Strengths and limitations of this study

- This survey is novel insofar as it examines a comprehensive set of factors linked to A&E attendance, including physical health, mental health, service access and socio-economic factors.
- Participants were recruited from a wide geographical area of disadvantaged neighbourhoods, which strengthens the representativeness of the survey findings.
- Households were approached at different times during the day to produce a less biased sample.
- Some of the most disadvantaged people may not be captured in the survey due to not having a fixed address.

For peer review only

INTRODUCTION

Accident and emergency (A&E) attendance rates are rapidly increasing in the UK and are particularly high in disadvantaged areas. This is leading to unsustainable pressure on the

For peer review only - http://bmjopen.bmj.com/site/about/guidelines.xhtml

NHS, with 20.5 million attendances reported in England from April 2015 to March 2016, compared to 19.5 million the previous year [1]. According to a King's Fund report, unnecessary A&E attendances could be reduced by between eight and 18% if patients attended a primary care service for their health concerns instead [2]. The predictors of A&E attendance are complex and involve multiple components. Whilst we know that A&E attendances are high in disadvantaged areas [3], the specific reasons for this are less clear. The prevalence of health problems is likely to be important; however, other factors may also play a role. Housing problems and employment status, for example, have been associated with high levels of A&E attendance, as have loneliness and low levels of social support [4,5]. Health service factors, including proximity to A&E and poor access to primary care, have also been linked to higher A&E attendance rates in past research [3, 6-11].

Previous studies on predictors of A&E attendance have been limited because they have not assessed the independent influence of the full range of potential risk factors, including morbidity, socio-economic factors, social support and proximity to A&E and primary care. In a recent study by Hull and colleagues [12], primary and secondary care data were linked with A&E attendance data in London. The data showed that multimorbidity, smoking, increased number of GP attendances, shorter distance to A&E, and age were all linked to visits to an A&E department. By comparing the most disadvantaged *versus* the least disadvantaged quintile, Hull and colleagues [12] reported a 52% higher A&E attendance in the most disadvantaged population sample.

The aim of this study was therefore to use data from a household health survey conducted in disadvantaged neighbourhoods in the North West of England to identify the

For peer review only - http://bmjopen.bmj.com/site/about/guidelines.xhtml

most important determinants of A&E attendance in disadvantaged populations to inform actions that could reduce demand for A&E services.

METHODS

Participants

The Northwest Coast area of England contains some of the most disadvantaged neighbourhoods in the country [13]. The Household Health Survey (HHS) of the National Institute for Health Research Collaboration for Leadership in Applied Health Research and Care in the North West Coast (NIHR CLAHRC NWC) was conducted between August 2015 and January 2016 to identify individual and neighbourhood level factors that contribute to inequalities in physical and mental health and to provide a baseline for evaluating neighbourhood level public heath interventions.

Twenty disadvantaged neighbourhoods were initially selected by participating local authorities. Each of the 10 Local Authorities that was part of the NIHR CLAHRC NWC partnership were asked to select a neighbourhood that would be involved in a number of public health initiatives and a comparator area with similar characteristics. The local authorities were asked to select these neighbourhood based on the following criteria: they (1) had a population between 5,000 and 10,000 residents; (2) had a deprivation index [13] in the bottom 15% of the national average and 3) had a coherent or shared sense of identity among residents. In addition the intervention areas needed to have a local community organisation that could support the implementation of the public health interventions. The selection of these neighbourhoods was at the discretion of the participating local authorities. The public health initiatives commenced in 10 of the

For peer review only - http://bmjopen.bmj.com/site/about/guidelines.xhtml

neighbourhoods after the survey data was collected. An additional sample (N=809) was also collected from 8 less disadvantaged neighbourhoods, however data from this sample was not used in this study.

Prior to the survey, ethical approval was obtained from the University of Liverpool (Ref: RETH000836). The survey fieldwork was conducted by BMG research and a pilot survey was completed with 36 residents from the disadvantaged neighbourhoods prior to the full data collection, which resulted in minor changes to survey documents. A random sample of households was then drawn from each of the disadvantaged neighbourhoods and one randomly selected respondent completed a face-to-face interview in each household (N=3,510). The survey combined information on a wide range of socioeconomic factors, mental and physical morbidities, and healthcare utilisation. Full details of the survey methodology are published elsewhere [14].

Variables

Our outcome variable was defined as the number of times respondents reported attending an A&E department over the previous twelve months. Measures of socioeconomic conditions included education (no qualifications, professional or vocational certificate, Degree or higher), employment (working/not working), financial hardship (doing well/getting by, struggling), change in financial circumstances (getting better or unchanged/worse than 12 months ago), housing quality (no problems / problems with cold, damp, or mold). Physical health was assessed with the four physical health dimensions of the EuroQuol five-dimension scale (EQ-5D) [15] for mobility, self-care, engagement in usual activities and pain (no problems/ some or severe problems). Mental health was

For peer review only

assessed using a series of validated instruments. Depression was measured using the nine-item Patient Health Questionnaire [16]. Anxiety was measured using the seven-item Generalized Anxiety Disorder scale [17]. Paranoia was measured using the persecution subscale of the persecution and deservedness scale (PaDS) for symptoms of paranoia [18]. Practical social support and social contact were assessed based on the level of agreement with the statements “If I needed help, there are people who would be there for me” and “If I wanted company or to socialize, there are people I can call on”. Response options for both items ranged from 1 = *definitely disagree* to 4 = *definitely agree*. (Further information on each of the survey variables is given in Web Appendix 1).

The proximity to A&E departments and GP practices was estimated using the Routino open source tool[1] to calculate the shortest road distance between the centre of each postcode and these health facilities. The average distance for all postcodes within each Lower layer Super Output Area (LSOA) level[2] was then estimated and linked to survey responses based on the LSOA in which the respondent lived.

Public involvement

Members of the public have been involved throughout the design and implementation of the survey, in addition to external partners and representatives from local authorities. This involvement included attending research and design meetings, providing feedback on study documents, as well as helping with the dissemination of findings. Public advisors have been recruited from the sampled disadvantaged areas and receive monetary reimbursement for their involvement.

[1] https://www.routino.org/uk/

[2] A Lower layer Super Output Area (LSOA) is a geographical area in the UK which is based on postcodes and clusters together groups of the population.

For peer review only - http://bmjopen.bmj.com/site/about/guidelines.xhtml

Data analysis

Data were analysed using Stata version 14. Because the A&E attendance variable comprised highly skewed (*S-W* = .39, $p < .001$) count data, we constructed a Poisson regression model. Standard errors were adjusted to account for the multi-stage nature of the survey sampling using the *svyset* commands in Stata [19] and the analysis was weighted for non-response. This model provided estimates of the Rate Ratio (RR) of A&E attendance associated with each variable, while holding all other variables constant in the model. We then used this model to calculate the Absolute Rate Differences (ARD) as the marginal effect of each of the variables whilst holding the other variables at their means. Demographic, socio-economic, health condition, disability, symptom, and healthcare access variables were entered into the model.

RESULTS

1,109 of the 3,510 survey respondents (31.6%) reported attending A&E at least once in the past 12 months. Among those, the number of attendances ranged from 1 to 95 (M=2.70, SD=4.90) and the overall rate of A&E attendance was 75 attendances per 100 population (95% CI 64, 87).

Table 1 shows the results of the Poisson regression model as well as descriptive statistics for the independent variables. Rate ratios (RR) and Absolute rate Differences (ARD) are reported alongside confidence intervals. Age was a significant demographic predictor of A&E attendances. After adjusting for other variables in the model the A&E attendance rate was three times higher amongst 18-24 year olds compared to people over

For peer review only - http://bmjopen.bmj.com/site/about/guidelines.xhtml

64 years of age (RR=2.72, 95%CI 1.33-5.53). In absolute terms, this was equivalent to an additional 60 attendances per 100 18-24 year olds compared to over 65 year olds, other factors being held constant (ARD = 60 per 100, 95% CI 10, 110). The health and disability variables predictive of A&E attendance included having a health condition as opposed to no health condition (RR=1.78, 95%CI 1.19-2.66, ARD = 28 per 100, 95%CI 10, 45), and having problems with self-care such as washing and dressing (RR=2.63, 95%CI 1.82-3.79; ARD = 71 per 100, 95%CI 34, 109). People with multiple morbidities were not more likely to attend A&E than people with only one condition. Of the socio-economic and environmental factors significantly associated with A&E attendance, being out of work increased risk of A&E attendance by 38% (RR=1.38, 95%CI 1.08-1.78; ARD = 16 per 100, 95%CI 4, 27) and living in a house with problems with cold, mould or damp increased risk of A&E attendance by 34% (RR=1.34, 95%CI 1.01 to 1.76; ARD = .14 per 100, 95%CI 1, 27). Surprisingly in this sample of people living in relatively disadvantaged neighbourhoods, higher levels of education were associated with higher risk of A&E attendance when controlling for other factors. Levels of social support and social contact did not predict A&E attendance.

Both healthcare access variables were significantly related to A&E attendance. Specifically, living further from an A&E department reduced the likelihood of attending an A&E service by seven percent per kilometre (RR = 0.93, 95%CI 0.89,0.97), and living further from a GP increased the likelihood of attending A&E by 46 percent per kilometre, after controlling for health status, socioeconomic and demographic factors (RR = 1.46, 95%CI 1.12, 1.90). Absolute Rate Differences indicated that for each kilometer closer the average person lived to an A&E department, they had 3 fewer A&E attendances per 100

For peer review only - http://bmjopen.bmj.com/site/about/guidelines.xhtml

population in the previous 12 months (ARD = -3, 95%CI -5, -2). Conversely, there were 18 more A&E attendances per 100 population for each kilometre further the average person lived from a GP practice (ARD = 18, 95%CI 6, 30).

DISCUSSION

This is one of the first surveys to collect data on socio-economic background, mental and physical health and health care utilisation, and to combine these factors with distance to health care services. Spanning a wide geographical area in one of the most disadvantaged areas of England [13], findings from the survey identify several key risk factors of A&E attendance rates, including young age, depression, high education, non-employment, poor housing, as well as longer distance from a GP and shorter distance to an A&E service.

Considering the increase in A&E attendance rates across England [1], particularly in disadvantaged areas [3], it is important to understand the reasons behind this rise and to design strategies to reduce health inequalities. A&E attendance was significantly higher for 18 to 24 year olds, when controlling for other factors, which has been found in previous work [20], and may be due to heightened rates of accidents, trauma and alcohol-related attendances at A&E [21], or higher use of A&E for primary care reasons in this age group. A limitation of our study is that we did not collect any information on the reasons for A&E attendance. Furthermore, A&E attendance has to be distinguished from admission to hospital, which we know is more prevalent in the elderly with long-term conditions [22].

Higher levels of education were linked to increased risks of attending A&E services in our disadvantaged sample. Considering that people from a low socioeconomic background are more likely to attend A&E services, as shown in this survey and supported

For peer review only - http://bmjopen.bmj.com/site/about/guidelines.xhtml

by other research [23-27], it is perhaps surprising to find those with a professional or degree level education to be up to twice as likely to access A&E when holding all other factors constant. It should be noted however that this is only the case when adjusting for other health and socioeconomic factors – the crude risk ratios for education were all not significantly different from 1. So it appears in our sample that where people with higher levels of education are living in disadvantaged areas and have similar levels of health problems as the rest of the population and are living in similar social circumstances they are more likely to attend A&E than there less educated peers. One possible reason for this may be that higher educated residents are more likely to recognise specific symptoms and become pro-active by seeking medical help. It is also plausible that people with higher qualifications have ended up living in disadvantaged neighbourhood, due to their poor health and this leads to increased A&E attendances. Indeed, depression as well as problems with self-care were independently associated with more A&E visits, supporting previous evidence of increased emergency attendances among people with mental health problems who are more likely to be "frequent users" [6, 28-29]. Thus, interventions that address mental health problems, particularly depression, and that provide greater support to people who have problems with self-care may be linked to concomitant declines in A&E attendance. However, the relationship between depression and A&E attendance should be interpreted with some caution because the rate ratio was found to be significant, while the absolute risk difference was not significant.

Being out of work and living in poor housing were important predictors of increased A&E attendance, above and beyond the effect of health status. This could be because these factors exacerbate existing conditions. Indeed, unemployment has been linked to

For peer review only - http://bmjopen.bmj.com/site/about/guidelines.xhtml

higher A&E attendances for asthma [30], whilst poor housing quality has been linked to more asthma-related symptoms [31] and worse mental health [32]. Controlling for these socioeconomic factors, the Index of Multiple Deprivation (IMD) was not independently associated with A&E attendance within this sample of disadvantaged areas. This indicates that much of the high level of A&E attendance found by others in disadvantaged areas [3] is possibly explained by differences in the prevalence of health conditions, high unemployment and poor housing. This lack of an effect related to deprivation may also be related to the limited variation in deprivation between the relatively deprived areas in our sample. Interventions that improve housing conditions and promote employment in disadvantaged communities should be integral to strategies to reduce demand for A&E services.

We found that longer distance from GP practices and shorter distance to A&E departments were significant predictors of A&E attendance. The findings are consistent with previous work on A&E proximity [8,9;12]; however, we additionally identified GP practice proximity as a unique determinant of A&E attendance. The results indicate that primary care access predicts A&E attendance over and above the effects of health status, socioeconomic status, and A&E access. The current trend of consolidating GP practices in fewer, larger health centres may therefore be contributing to increases in A&E attendances. This trend may also be linked to ease of access, in that patients may be unable to get a GP appointment and therefore attend an A&E service instead. Future approaches should be cognisant of primary care service placement and access. Reduced demand for A&E may be achieved via improved public transport links that consider primary care locations, or via strategically positioning primary care services in areas with high A&E

For peer review only - http://bmjopen.bmj.com/site/about/guidelines.xhtml

For peer review only

attendance rates. It would be of interest in future research to examine whether factors such as transport links or appointment availability independently predict A&E attendance or, in the case of public transport links, explain the relationship between distance to services and service usage.

One recently conducted retrospective study has also explored the socioeconomic predictors of A&E attendance [12], showing that A&E attendance increased with increasing numbers of health conditions, in addition to risk of A&E attendance increase with smoking, GP attendance, being housebound, and age. However, the study differed in several aspects from the household health survey. On the one hand, the study by Hull and colleagues [12] did not measure the effects of proximity to GP services. In addition, our household health survey contains a rich set of mental health, health related quality of life and socioeconomic variables, whereas the London-based assessment of primary and secondary care data only employs the IMD as a measure of social deprivation, having no information on for example housing quality and health measures were limited to numbers of conditions. In our study A&E attendance did not increase with increasing numbers of health conditions, this may be because the number of health conditions a person has is not in itself a risk for A&E attendance – but this is just a marker of people with poorer mental health and health related quality of life.

The findings from this survey should be considered in light of a few limitations. The cross-sectional nature of the survey only allowed investigation of the predictors of A&E attendance at one point in time. Moreover, this survey was based on self-reports, and thus participants had to remember whether they had attended an A&E department, and how often, in the past 12 months. This could be avoided by referring to Hospital Episodes

For peer review only - http://bmjopen.bmj.com/site/about/guidelines.xhtml

Statistics data from the National Health Service. The survey also purposefully recruited residents from some of the most disadvantaged areas within England and the Northwest Coast, and selected only neighbourhoods that met criteria that included methodological (e.g., large population and shared neighbourhood identity) and practical (e.g., infrastructure for interventions) constraints. Thus, the findings may not generalise to other areas that did not meet these criteria. Lastly, people without a fixed address who may be living in extremely disadvantaged circumstances were probably not captured by our data.

Conclusions

We conducted a cross-sectional analysis of A&E attendance risk factors in a large sample of UK residents in disadvantaged neighbourhoods. To our knowledge, this is the most comprehensive examination of diverse socioeconomic determinants of A&E attendance, and the first to examine GP proximity alongside A&E proximity. Housing quality, employment status, self-care difficulties, depression, and proximity to both primary and emergency services were identified as the most important determinants of A&E attendance. The findings highlight the need for multi-pronged approaches to reduce A&E attendances that address socioeconomic inequalities, such as employment and housing quality, but also structural issues such as access to primary care. Key policy suggestions likely to reduce A&E attendance based on these findings include: strategic placement of — and better transport links to — primary care services, better housing conditions, increased employment and improved support for self-care and public mental health. Improving housing conditions has shown short-term benefits on the health of residents, without having evaluated A&E attendance rates [31; 33]. To date, findings on the effect of

For peer review only

For peer review only - http://bmjopen.bmj.com/site/about/guidelines.xhtml

improving primary care access interventions on reducing A&E attendance appear inconclusive [34], suggesting the need for further implementation strategies to improve primary care access. Strategies to reduce strain on health services need to address the social and economic factors that underpin demand for healthcare and enhance access to primary care.

For peer review only

Funding

This work was supported by the National Institute for Health Research, Collaboration for Leadership and Health Research and Care North West Coast (NIHR CLAHRC NWC). The views expressed are those of the author(s) and not necessarily those of the NHS, the NIHR or the Department of Health.

Competing interests

We have read and understood BMJ policy on declaration of interests and declare the following: All authors have completed the ICMJE uniform disclosure form. The authors have no financial relationships with any organisations that might have an interest in the submitted work in the previous three years, no other relationships or activities that could appear to have influenced the submitted work.

Author Contributions

CG and JMI jointly wrote the article, and JMI and BB performed statistical analysis. KD, MP, MG, JD, FW, WS, AA, and BB edited and provided feedback on drafts of the manuscript and approved the final version.

Research ethics

The research was approved by the University of Liverpool Committee on Research Ethics (Ref: RETH00836). Participants provided informed consent before completing the study.

Data sharing statement

For peer review only - http://bmjopen.bmj.com/site/about/guidelines.xhtml

For peer review only

The dataset from the North West Coast Household Health Survey will be made publicly available after an embargo period.

For peer review only

For peer review only - http://bmjopen.bmj.com/site/about/guidelines.xhtml

References

[1] NHS Digital, 2017. Hospital Accident and Emergency Activity 2015-2016. Health and Social Care Information Centre, London: NHS Digital.

[2] Tian Y. 2012. Data briefing: emergency hospital admissions for ambulatory care-sensitive conditions. King's Fund report. https://www.kingsfund.org.uk/sites/default/files/field/field_publication_file/data-briefing-emergency-hospital-admissions-for-ambulatory-care-sensitive-conditions-apr-2012.pdf (accessed 28 Sep 2017).

[3] Scantlebury R, Rowlands G, Durbaba S, Schofield P, Sidhu K, Ashworth M. Socioeconomic deprivation and accident and emergency attendances: cross-sectional analysis of general practice in England. Br J Gen Pract 2015;65(639):e649-e654.

[4] Geller J, Janson P, McGovern E, Valdini A. Loneliness as a Predictor of Hospital Emergency Department Use. The Journal of Family Practice 1999;48(10): 801–4.

[5] Molloy GJ, McGee HM, O'Neill D, Conroy RM. Loneliness and Emergency and Planned Hospitalizations in a Community Sample of Older Adults. *Journal of the American Geriatrics Society* 2010;58(8): 1538–41.

[6] Daniels J, Osborn M, Davis C. Better safe than sorry? Frequent attendance in a hospital emergency department: an exploratory study. Br J Pain 2017; doi:10.1177/2049463717720635

[7] Hull SA, Jones IR, Moser K. Factors influencing the attendance rate at accident and emergency departments in East London: The contributions of practice organization, population characteristics and distance. J Health Services Research Policy 1997;2(1):6-13.

[8] Paul P, Heng BH, Seow E, Molina J, Tay SY. Predictors of frequent attenders of emergency department at an acute general hospital in Singapore. Emergency Medical Journal 2010;27:805-805.

[9] Rudge GM, Mohammed MA, Fillingham SC, et al. The Combined Influence of Distance and Neighbourhood Deprivation on Emergency Department Attendance in a Large English Population: A Retrospective Database Study. PLOS One 2013; doi: 10.1371/journal.pone.0067943

[10] Takahashi PY, Ryu E, Hathcock MA, et al. A novel housing-based socioeconomic measure predicts hospitalization and multiple chronic conditions in a community population. J Epidemiology Community Health 2016;70(3):286-291.

[11] Tammes P, Morris RW, Brangan E, et al. Exploring the relationship between general practice characteristics, and attendance at walk-in centres, minor injuries units and EDs in England 2012/2013: a cross-sectional study. BMJ 2017;33:702-708.

[12] Hull SA, Homer K, Boomla K, Robson J, Ashworth M. Population and patient factors affecting emergency department attendance in London: retrospective cohort analysis of linked primary and secondary care records. Br J Gen Pract 2018; doi: 10.3399/bjgp18X694397

[13] Department for Communities and Local Government. The English Indices of Deprivation 2015: technical report. London: 2015.

For peer review only - http://bmjopen.bmj.com/site/about/guidelines.xhtml

[14] McIntyre J, Wickham S, Barr B, Bentall RP. Social Identity and Psychosis: Associations and Psychological Mechanisms. Schizophrenia Bulletin 2017, doi: 10.1093/schbul.sbx110.
[15] Herdman M, Gudex C, Lloyd A, Janssen MF, Kind P, Parkin D, Bonsel G, Badia X. Development and preliminary testing of the new five-level version of EQ-5D (EQ-5D-5L). Quality of Life Research 2011;20(10):1727-1736.
[16] Kroenke K, Spitzer RL, Williams JBW. The PHQ-9 – Validity of a Brief Depression Severity Measure. J Gen Intern Med 2001;16(9):606-613.
[17] Spitzer RL, Kroenke K, Williams JBW, Loewe B. A brief measure for assessing Generalized Anxiety Disorder – The GAD-7. Arch Intern Med 2006;166:1092-1097.
[18] Melo S, Corcoran R, Shryane N, Bentall RP. The persecution and deservedness scale. Psychol Psychother 2009;82:247-260.
[19] StataCorp. 2015. Stata Survey Data Reference Manual. College Station, TX: StataCorp LLC.
[20] Health and Social Care Information Centre. *Hospital Accident and Emergency Activity: 2015-16*. NHS Digital, England: 2017.
[21] Parkinson K, Newbury-Birch D, Phillipson A, et al. Prevalence of alcohol related attendance at an inner city emergency department and its impact: a dual prospective and retrospective cohort study. Emergency Medicine Journal 2016;33:187-193.
[22] King's Fund. *Long-term conditions and multi-morbidity*. Last accessed 15th January 2018: https://www.kingsfund.org.uk/projects/time-think-differently/trends-disease-and-disability-long-term-conditions-multi-morbidity
[23] Krieg C, Hudon C, Chouinard MC, Dufour I. Individual predictors of frequent emergency department use: a scoping review. BMC Health Serv Res 2016; 16:594.
[24] McKetin R, Degenhardt L, Shanahan M, et al. Health service utilization attributable to methamphetamine use in Australia: Patterns, predictors and national impact. Drug Alc Rev 2018;37(2):196-204.
[25] Pines JM, Buford K. Predictors of frequent emergency department utilization in Southeastern Pennsylvania. J Asthma. 2006;43(3):219–23.
[26] Sun BC, Burstin HR, Brennan TA. Predictors and outcomes of frequent emergency department users. Acad Emerg Med. 2003;10(4):320–8.
[27] Zuckerman S, Shen YC. Characteristics of occasional and frequent emergency department users: do insurance coverage and access to care matter? Med Care. 2004;42(2):176–82.
[28] Keene J, Rodriguez J. Are mental health problems associated with use of Accident and Emergency and health-related harm? European J Public Health 2007;17(4):387-393.
[29] Ingram WM, Weston C, Ritchie MD, Larson S. Depression linked to frequent emergency department use in large 10-year retrospective analysis of an integrated health care system. bioRxiv 2017; doi:10.1101/115238
[30] Forbes L, Harvey S, Newson R, et al. Risk Factors for Accident and Emergency (A&E) Attendance for Asthma in Inner City Children. Thorax 2007;62(10):855–60.
[31] Barton A, Basham M, Foy C, Buckingham K, Somerville M. The Watcombe Housing Study: the short term effect of improving housing conditions on the health of residents. Journal of Epidemiology Community Health 2007;61(9):771-777.

For peer review only - http://bmjopen.bmj.com/site/about/guidelines.xhtml

[32] Evans GW, Wells NM, Chan HYE, Saltzman H. Housing quality and mental health. J Consulting Clin Psychol 2000;68(3):526-530.
[33] Woodfine L, Neal RD, Bruce N, Edwards RT, Linck P, Mullock L, et al. Enhancing ventilation in homes of children with asthma: pragmatic randomized controlled trial. Br J Gen Pract 2011;61(592):e727-e732.
[34] Ismail SA, Gibbons DC, Gnani S. Reducing inappropriate accident and emergency department attendances: a systematic review of primary care service interventions. Br J Gen Pract 2013;63(617):e813-e820.

For peer review only

For peer review only - http://bmjopen.bmj.com/site/about/guidelines.xhtml

Table 1. Determinants of number of A&E attendances over the previous 12 months (n=3,510)

Socioeconomic factors	Rate Ratio of A&E attendance	Rate Ratio 95% CI	Absolute Risk Difference of A&E attendance	Absolute Risk Difference 95% CI	Unadjusted Rate Ratios Ratio (95% CI)	N (%)	M (SD)
**Demographics**							
Age (65+)						858 (24.5)	-
18-24	2.72**	1.33, 5.53	.60*	.10, 1.10	1.15 (.70,1.88)	368 (10.5)	-
25-44	1.43	.95, 2.16	.15	-.01, .31	.83 (.62,1.12)	1226 (34.9)	-
45-64	1.21	.83, 1.77	.07	-.07, .21	1.18 (.81,1.72)	1057 (30.1)	-
Gender (female)	.90	.69, 1.18	-.05	-.18, .08	.89 (.66,1.20)	2029 (57.8)	-
Ethnicity (BME)	.60	.35, 1.04	-.20*	-.37, -.03	.40 (.24,.67)	366 (10.5)	-
**Socioeconomic status**							
Education (No qualifications)						1516 (43.3)	-
Professional/vocational certificate	1.58**	1.17, 2.14	.20**	.07, .33	1.20 (.88, 1.63	1579 (45.1)	-
Degree or higher	2.00*	1.15, 3.48	.35*	.02, .67	1.21 (.71, 2.07)	405 (11.6)	-
Non-employment (Not in paid/self-employment)	1.38*	1.08, 1.78	.16**	.04, .27	2.20 (1.65, 2.93)	2150 (61.3)	-
Index of Multiple Deprivation	.99	.99, 1.00	-.003	-.01, .001	.99 (.99, 1.01)	-	47.20 (16.90)
Financial struggle ("Doing well")						716 (20.4)	-
"Getting by"	1.00	.72, 1.40	<.001	-.16, .16	1.33 (.96, 1.86)	2307 (65.8)	-
"Struggling"	1.22	.75, 1.98	.10	-.16, .36	2.94 (1.78, 4.85)	481 (13.7)	-
Financial situation worse than 12 months ago	.99	.78, 1.26	-.004	-.12, .11	1.49 (1.07, 2.09)	578 (16.6)	-
**Housing quality**							
Problems with condensation/mold/ temperature	1.34*	1.01, 1.76	.14*	.01, .27	1.73 (1.26, 2.38)	1138 (33.7)	-
**Health status**							
Mobility problems (EQ5D)	1.24	.84, 1.83	.11	-.10, .32	3.54 (2.64, 4.76)	930 (73.5)	-
Self-care problems (EQ5D)	2.63***	1.82, 3.79	.71***	.34, 1.09	5.58 (3.85, 8.09)	381 (10.9)	-
Problems engaging in usual activities (EQ5D)	1.43*	1.01, 2.03	.19	-.02, .40	4.17 (3.12, 5.57)	852 (24.3)	-
Pain (EQ5D)	1.24	.90, 1.71	.11	-.05, .27	3.30 (2.50, 4.36)	1325 (37.8)	-
1 condition (base category: no condition)	1.78**	1.19, 2.66	.28**	.10, .45	3.19 (2.38, 4.27)	857 (24.4)	-
>1 condition (base category: 1 condition)	1.02	.67, 1.55	.01	-.19, .21	2.80 (2.08, 3.77)	1256 (35.8)	-

For peer review only

**Mental Health**	1.36*	1.01, 1.83	.15	-.003, .30	2.02 (1.72, 2.37)	-	
Depression (PHQ-9)	.86	.65, 1.14	-.07	-.21, .06	1.75 (1.47, 2.07)	-	1.56 (.68)
Anxiety (GAD-7)	1.04	.89, 1.21	.02	-.06, .09	1.41 (1.21, 1.65)	-	1.54 (.75)
Paranoia (PaDS-5)							2.01 (.89)
**Social support**	.93	.47, 1.85	-.03	-.36, .29	.69 (.30, 1.59)	3302 (94.3)	
Practical support	1.08	.70, 1.70	.04	-.17, .25	.64 (.31, 1.31)	3286 (94.0)	-
Social contact							-
**Healthcare access**	1.46**	1.12, 1.90	.18**	.06, .30	1.21 (.96, 1.53)		
Distance to GP (km)	.93***	.89, .97	-.03***	-.05, -.02	.95 (.91, .99)		.99 (.66)
Distance to A&E (km)							7.12(4.21)

* $p < .05$, ** $p < .01$, *** $p < .001$

NOTE: Risk Ratios and Absolute Risk Differences are adjusted for all other variables in the model. BME= Black and Minority Ethnic; EQ5D= Euroqol 5 dimensions; a measure of generic health status; GAD-7=Generalised Anxiety Disorder Assessment; PHQ-9=Patient Health Questionnaire; PaDS-5= Persecution and Deservedness Scale

For peer review only

Appendix A: Table of Measures

Variable	Source	Description/Example	Original scoring	Recoding for analysis
Age	Office for National Statistics	N/A	1 = Under 16 years<br>2 = 16-17 years<br>3 = 18-24 years<br>4 = 25-34 years<br>5 = 35-44 years<br>6 = 45-54 years<br>7 = 55-64 years<br>8 = 65-74 years<br>9 = 75+ years	1 = 18-24 years<br>2 = 25-44 years<br>3 = 45-64 years<br>4 = 65+ years
Sex	Office for National Statistics	N/A	1 = Male<br>2 = Female<br>3 = Other	1 = Male<br>2 = Female
Ethnicity	Office for National Statistics	N/A	1 = English / Welsh / Scottish / Northern Irish / British<br>2 = Irish<br>3 = Gypsy or Irish Traveller<br>4 = Any other White background, *please specify*<br>5 = White and Black Caribbean<br>6 = White and Black African<br>7 = White and Asian<br>8 = Any other Mixed / Multiple ethnic background, *please specify*<br>9 = Indian<br>10 = Pakistani<br>11 = Bangladeshi<br>12 = Chinese<br>13 = Any other Asian background, *please specify*<br>14 = African<br>15 = Caribbean<br>16 = Any other Black / African / Caribbean background, *please specify*<br>17 = Arab<br>95 = Any other group, please specify	0 = White<br>1 = BME
Non-	Office for National	N/A	1 = Going to school or college full time (including on vacation)	0 = employed

For peer review only

employment	Statistics		2 = In paid employment or self employed (or temporarily away)<br>3 = On a Government scheme for employment training<br>4 = Doing unpaid work for a business that you own, or that a relative owns<br>5 = Waiting to take up paid work already obtained<br>6 = Looking for paid work or a Government training scheme<br>7 = Intending to look for work but prevented by temporary sickness or injury<br>8 = Permanently unable to work because of long-term sickness or disability<br>9 = Retired from paid work<br>10 = Looking after the home or family<br>95 = Doing something else, specify	1 = not employed
Education	Office for National Statistics	Do you have any educational qualifications for which you received a certificate?<br><br>Do you have any professional, vocational or other work-related qualifications for which you received a certificate?<br><br>What is your highest qualification?	1 = Yes, 2 = No<br><br>1 = Yes, 2 = No<br><br>1 = At degree level or above, 2 = Another kind of qualification	1 = No qualifications<br>2 = Professional or vocational qualification<br>3 = Degree or higher
Housing quality	English Housing Survey	During the winter months, does condensation form on the windows or walls of any room in your home apart from the bathrooms or toilets?<br><br>During the winter months, are there patches of mould or fungus in any room in your home, apart from bathrooms or toilets?	1 = Yes, 2 = No, 3 = Spontaneous: don't know<br><br>1 = Yes, 2 = No, 3 = Spontaneous: don't know	0 = No problems<br>1 = One problem<br>2 = Two problems<br>3 = Problems with all three issues

For peer review only

		During the cold winter weather, can you normally keep comfortably warm in your living room?	1 = Yes, 2 = No, 3 = Spontaneous: don't know	
Financial struggle	Wealth and Assets Survey	How well would you say your household is managing financially these days?	1 = Doing well<br>2 = Getting by<br>3 = Struggling	N/A
Financial Situation	Wealth and Assets Survey	Would you say your household is better off or worse off financially than you were a year ago?	1 = Better off<br>2 = About the same<br>3 = Worse off<br>4 = Don't know	1 = Better off<br>2 = About the same<br>3 = Worse off
Index of Multiple Deprivation (IMD)	Office of National Statistics	Measure of deprivation at level of Lower Level Super Output Area (LSOA) across seven domains: Income, Employment, Education, Health and Disability, Crime, Housing, ad Living Environment.	N/A	N/A
Physical Health	EuroQual Five Dimensional Scale (EQ-5D)	Standardised instrument that assesses problems with mobility, self-care, engagement in usual activities, and pain.	1 = No problems<br>2 = Some Problems/Moderate problems<br>3 = Extreme problems/Unable	0 = No problems<br>1 = Some/Severe problems
Multimorbidity	Psychiatric Morbidity Survey	Have you ever had any of [these health conditions] over the past 12 months?	1 = Cancer<br>2 = Diabetes<br>3 = Epilepsy/fits<br>4 = Migraine or other frequent headaches<br>5 = Dementia or Alzheimer's disease<br>6 = Any mental health issue<br>7 = Cataracts / eyesight problems (even if corrected with glasses or contacts)<br>8 = Ear/hearing problems (even if corrected with a hearing aid)<br>9 = Stroke	*At least one condition*<br><br>0 = No conditions<br>1 = One condition<br><br>*Multimorbidity*<br><br>0 = One condition<br>1 = More than one condition

For peer review only

			10 = Heart attack/angina<br>11 = High blood pressure<br>12 = Bronchitis/emphysema<br>13 = Asthma<br>14 = Allergies<br>15 = Stomach ulcer or other digestive problems<br>16 = Liver problems<br>17 = Bowel/colon problems<br>18 = Bladder problems/incontinences<br>19 = Arthritis<br>20 = Bone, back joint or muscle problems<br>21 = Gout<br>22 = Skin problems<br>95 = Other, please specify<br>96 = None of these	
Depression	Patient Health Questionnaire (PHQ-9)	Assesses how often participants had been bothered by pro problems such as "Feeling down, depressed, or hopeless" over the past two weeks.	1 = Not at all<br>2 = Several days<br>3 = More than half the days<br>4 = Nearly every day	N/A
Anxiety	Generalised Anxiety Disorder Questionnaire (GAD-7)	Assesses frequency of events such as "Being so restless that it is hard to sit still" over the past two weeks.	1 = Not at all<br>2 = Several days<br>3 = More than half the days<br>4 = Nearly every day	N/A
Paranoia	Five-item Persecution and Deservedness Scale (PaDS-5)	Assesses the extent to which people are suspicious of others' intentions. Participants rate their level of agreement with statements such as "I'm often suspicious of other people's intentions towards me."	1 = Strongly disagree<br>2 = Disagree<br>3 = Neither agree or disagree<br>4 = Agree<br>5 = Strongly agree	N/A
Social Support	Community Life Survey	Assesses the extent to which participants agree they receive *practical support* ("If I	1 = Definitely agree<br>2 = Tend to agree<br>3 = Tend to disagree	0 = Disagree<br>1 = Agree

For peer review only

		needed help, there are people who would be there for me") and *social contact* (If I wanted company or to socialise, there are people I can call on")	4 = Definitely disagree<br>5 = Don't know	
Healthcare Access	N/A	The *proximity to A&E departments* and *proximity to GP practices* was estimated using the Routino open source tool (Routino.org.uk) to calculate the shortest road distance in kilometres between the centre of each postcode and these health facilities. The average distance for all postcodes within each Lower layer Super Output Area (LSOA) level was then estimated and linked to survey responses based on the LSOA in which the respondent lived.	N/A	N/A
Healthcare usage	SANAD II trial	Have you been to a hospital casualty/A&E/ urgent care department in the past 12 months?	1=Yes; 2=No<br>If yes, please specify total number	N/A

For peer review only

STROBE Statement—checklist of items that should be included in reports of observational studies

	Item No	Recommendation	Page in manuscript
**Title and abstract**	1	(*a*) Indicate the study's design with a commonly used term in the title or the abstract	
		(*b*) Provide in the abstract an informative and balanced summary of what was done and what was found	Abstract
**Introduction**			
Background/rationale	2	Explain the scientific background and rationale for the investigation being reported	3
Objectives	3	State specific objectives, including any prespecified hypotheses	4
**Methods**			
Study design	4	Present key elements of study design early in the paper	4
Setting	5	Describe the setting, locations, and relevant dates, including periods of recruitment, exposure, follow-up, and data collection	4
Participants	6	(*a*) *Cohort study*—Give the eligibility criteria, and the sources and methods of selection of participants. Describe methods of follow-up *Case-control study*—Give the eligibility criteria, and the sources and methods of case ascertainment and control selection. Give the rationale for the choice of cases and controls *Cross-sectional study*—Give the eligibility criteria, and the sources and methods of selection of participants	4
		(*b*) *Cohort study*—For matched studies, give matching criteria and number of exposed and unexposed *Case-control study*—For matched studies, give matching criteria and the number of controls per case	
Variables	7	Clearly define all outcomes, exposures, predictors, potential confounders, and effect modifiers. Give diagnostic criteria, if applicable	5
Data sources/ measurement	8*	For each variable of interest, give sources of data and details of methods of assessment (measurement). Describe comparability of assessment methods if there is more than one group	*5*
Bias	9	Describe any efforts to address potential sources of bias	
Study size	10	Explain how the study size was arrived at	5
Quantitative variables	11	Explain how quantitative variables were handled in the analyses. If applicable, describe which groupings were chosen and why	5
Statistical methods	12	(*a*) Describe all statistical methods, including those used to control for confounding	5
		(*b*) Describe any methods used to examine subgroups and interactions	
		(*c*) Explain how missing data were addressed	
		(*d*) *Cohort study*—If applicable, explain how loss to follow-up was addressed *Case-control study*—If applicable, explain how matching of cases and controls was addressed *Cross-sectional study*—If applicable, describe analytical methods taking account of sampling strategy	

For peer review only

For peer review only - http://bmjopen.bmj.com/site/about/guidelines.xhtml

(*e*) Describe any sensitivity analyses

Continued on next page

For peer review only

Results			Page in the manuscript
Participants	13*	(a) Report numbers of individuals at each stage of study—eg numbers potentially eligible, examined for eligibility, confirmed eligible, included in the study, completing follow-up, and analysed	6
		(b) Give reasons for non-participation at each stage	
		(c) Consider use of a flow diagram	
Descriptive data	14*	(a) Give characteristics of study participants (eg demographic, clinical, social) and information on exposures and potential confounders	6
		(b) Indicate number of participants with missing data for each variable of interest	
		(c) *Cohort study*—Summarise follow-up time (eg, average and total amount)	
Outcome data	15*	*Cohort study*—Report numbers of outcome events or summary measures over time	
		Case-control study—Report numbers in each exposure category, or summary measures of exposure	
		Cross-sectional study—Report numbers of outcome events or summary measures	*6*
Main results	16	(*a*) Give unadjusted estimates and, if applicable, confounder-adjusted estimates and their precision (eg, 95% confidence interval). Make clear which confounders were adjusted for and why they were included	6
		(*b*) Report category boundaries when continuous variables were categorized	
		(*c*) If relevant, consider translating estimates of relative risk into absolute risk for a meaningful time period	
Other analyses	17	Report other analyses done—eg analyses of subgroups and interactions, and sensitivity analyses	
**Discussion**			
Key results	18	Summarise key results with reference to study objectives	7-9
Limitations	19	Discuss limitations of the study, taking into account sources of potential bias or imprecision. Discuss both direction and magnitude of any potential bias	9
Interpretation	20	Give a cautious overall interpretation of results considering objectives, limitations, multiplicity of analyses, results from similar studies, and other relevant evidence	7-9
Generalisability	21	Discuss the generalisability (external validity) of the study results	7-10
**Other information**			
Funding	22	Give the source of funding and the role of the funders for the present study and, if applicable, for the original study on which the present article is based	11

*Give information separately for cases and controls in case-control studies and, if applicable, for exposed and unexposed groups in cohort and cross-sectional studies.

For peer review only

Note: An Explanation and Elaboration article discusses each checklist item and gives methodological background and published examples of transparent reporting. The STROBE checklist is best used in conjunction with this article (freely available on the Web sites of PLoS Medicine at http://www.plosmedicine.org/, Annals of Internal Medicine at http://www.annals.org/, and Epidemiology at http://www.epidem.com/). Information on the STROBE Initiative is available at www.strobe-statement.org.

For peer review only

For peer review only - http://bmjopen.bmj.com/site/about/guidelines.xhtml

BMJ Open

What are the social predictors of Accident & Emergency attendance in disadvantaged neighbourhoods? Results from a cross-sectional household health survey in the North West of England

Journal:	*BMJ Open*
Manuscript ID	bmjopen-2018-022820.R2
Article Type:	Research
Date Submitted by the Author:	30-Jul-2018
Complete List of Authors:	Giebel, Clarissa; University of Liverpool, Institute of Psychology, Health & Society; CLAHRC NWC<br>McIntyre, Jason; University of Liverpool, Institute of Psychology, Health & Society<br>Daras, Konstantinos ; University of Liverpool, Geographic Data Sciences Lab<br>Gabbay, Mark; University of Liverpool, Institute of Psychology, Health & Society; CLAHRC NWC<br>Downing, Jennifer; University of Liverpool, Department of Molecular and Clinical Pharmacology<br>Pirmohamed, Munir; The University of Liverpool, Department of Pharmacology; CLAHRC NWC<br>Walker, Fran; CLAHRC NWC<br>Sawicki, Woiciech; Royal Liverpool and Broadgreen University Hospitals NHS Trust<br>Alfirevic, Ana; University of Liverpool, Institute of Translational Medicine; CLAHRC NWC<br>Barr, Ben ; University of Liverpool, Institute of Psychology, Health & Society; CLAHRC NWC
**Primary Subject Heading**:	Public health
Secondary Subject Heading:	Health services research
Keywords:	accident and emergency, service access, Depression & mood disorders < PSYCHIATRY, deprivation, housing quality
Note: The following files were submitted by the author for peer review, but cannot be converted to PDF. You must view these files (e.g. movies) online.	
Predictors_of_AE_Attendance_Revisions_FINAL_clean copy.docx	

For peer review only

Appendix A: Table of Measures

Variable	Source	Description/Example	Original scoring	Recoding for analysis
Age	Office for National Statistics	N/A	1 = Under 16 years<br>2 = 16-17 years<br>3 = 18-24 years<br>4 = 25-34 years<br>5 = 35-44 years<br>6 = 45-54 years<br>7 = 55-64 years<br>8 = 65-74 years<br>9 = 75+ years	1 = 18-24 years<br>2 = 25-44 years<br>3 = 45-64 years<br>4 = 65+ years
Sex	Office for National Statistics	N/A	1 = Male<br>2 = Female<br>3 = Other	1 = Male<br>2 = Female
Ethnicity	Office for National Statistics	N/A	1 = English / Welsh / Scottish / Northern Irish / British<br>2 = Irish<br>3 = Gypsy or Irish Traveller<br>4 = Any other White background, *please specify*<br>5 = White and Black Caribbean<br>6 = White and Black African<br>7 = White and Asian<br>8 = Any other Mixed / Multiple ethnic background, *please specify*<br>9 = Indian<br>10 = Pakistani<br>11 = Bangladeshi<br>12 = Chinese<br>13 = Any other Asian background, *please specify*<br>14 = African<br>15 = Caribbean<br>16 = Any other Black / African / Caribbean background, *please specify*<br>17 = Arab<br>95 = Any other group, please specify	0 = White<br>1 = BME
Non-	Office for National	N/A	1 = Going to school or college full time (including on vacation)	0 = employed

For peer review only

employment	Statistics		2 = In paid employment or self employed (or temporarily away)<br>3 = On a Government scheme for employment training<br>4 = Doing unpaid work for a business that you own, or that a relative owns<br>5 = Waiting to take up paid work already obtained<br>6 = Looking for paid work or a Government training scheme<br>7 = Intending to look for work but prevented by temporary sickness or injury<br>8 = Permanently unable to work because of long-term sickness or disability<br>9 = Retired from paid work<br>10 = Looking after the home or family<br>95 = Doing something else, specify	1 = not employed
Education	Office for National Statistics	Do you have any educational qualifications for which you received a certificate?<br><br>Do you have any professional, vocational or other work-related qualifications for which you received a certificate?<br><br>What is your highest qualification?	1 = Yes, 2 = No<br><br>1 = Yes, 2 = No<br><br>1 = At degree level or above, 2 = Another kind of qualification	1 = No qualifications<br>2 = Professional or vocational qualification<br>3 = Degree or higher
Housing quality	English Housing Survey	During the winter months, does condensation form on the windows or walls of any room in your home apart from the bathrooms or toilets?<br><br>During the winter months, are there patches of mould or fungus in any room in your home, apart from bathrooms or toilets?	1 = Yes, 2 = No, 3 = Spontaneous: don't know<br><br>1 = Yes, 2 = No, 3 = Spontaneous: don't know	0 = No problems<br>1 = One problem<br>2 = Two problems<br>3 = Problems with all three issues

For peer review only

		During the cold winter weather, can you normally keep comfortably warm in your living room?	1 = Yes, 2 = No, 3 = Spontaneous: don't know	
Financial struggle	Wealth and Assets Survey	How well would you say your household is managing financially these days?	1 = Doing well<br>2 = Getting by<br>3 = Struggling	N/A
Financial Situation	Wealth and Assets Survey	Would you say your household is better off or worse off financially than you were a year ago?	1 = Better off<br>2 = About the same<br>3 = Worse off<br>4 = Don't know	1 = Better off<br>2 = About the same<br>3 = Worse off
Index of Multiple Deprivation (IMD)	Office of National Statistics	Measure of deprivation at level of Lower Level Super Output Area (LSOA) across seven domains: Income, Employment, Education, Health and Disability, Crime, Housing, ad Living Environment.	N/A	N/A
Physical Health	EuroQual Five Dimensional Scale (EQ-5D)	Standardised instrument that assesses problems with mobility, self-care, engagement in usual activities, and pain.	1 = No problems<br>2 = Some Problems/Moderate problems<br>3 = Extreme problems/Unable	0 = No problems<br>1 = Some/Severe problems
Multimorbidity	Psychiatric Morbidity Survey	Have you ever had any of [these health conditions] over the past 12 months?	1 = Cancer<br>2 = Diabetes<br>3 = Epilepsy/fits<br>4 = Migraine or other frequent headaches<br>5 = Dementia or Alzheimer's disease<br>6 = Any mental health issue<br>7 = Cataracts / eyesight problems (even if corrected with glasses or contacts)<br>8 = Ear/hearing problems (even if corrected with a hearing aid)<br>9 = Stroke	*At least one condition*<br><br>0 = No conditions<br>1 = One condition<br><br>*Multimorbidity*<br><br>0 = One condition<br>1 = More than one condition

For peer review only

			10 = Heart attack/angina<br>11 = High blood pressure<br>12 = Bronchitis/emphysema<br>13 = Asthma<br>14 = Allergies<br>15 = Stomach ulcer or other digestive problems<br>16 = Liver problems<br>17 = Bowel/colon problems<br>18 = Bladder problems/incontinences<br>19 = Arthritis<br>20 = Bone, back joint or muscle problems<br>21 = Gout<br>22 = Skin problems<br>95 = Other, please specify<br>96 = None of these	
Depression	Patient Health Questionnaire (PHQ-9)	Assesses how often participants had been bothered by pro problems such as “Feeling down, depressed, or hopeless” over the past two weeks.	1 = Not at all<br>2 = Several days<br>3 = More than half the days<br>4 = Nearly every day	N/A
Anxiety	Generalised Anxiety Disorder Questionnaire (GAD-7)	Assesses frequency of events such as “Being so restless that it is hard to sit still” over the past two weeks.	1 = Not at all<br>2 = Several days<br>3 = More than half the days<br>4 = Nearly every day	N/A
Paranoia	Five-item Persecution and Deservedness Scale (PaDS-5)	Assesses the extent to which people are suspicious of others’ intentions. Participants rate their level of agreement with statements such as “I’m often suspicious of other people’s intentions towards me.”	1 = Strongly disagree<br>2 = Disagree<br>3 = Neither agree or disagree<br>4 = Agree<br>5 = Strongly agree	N/A
Social Support	Community Life Survey	Assesses the extent to which participants agree they receive *practical support* (“If I	1 = Definitely agree<br>2 = Tend to agree<br>3 = Tend to disagree	0 = Disagree<br>1 = Agree

For peer review only

		needed help, there are people who would be there for me") and *social contact* (If I wanted company or to socialise, there are people I can call on")	4 = Definitely disagree<br>5 = Don't know	
Healthcare Access	N/A	The *proximity to A&E departments* and *proximity to GP practices* was estimated using the Routino open source tool (Routino.org.uk) to calculate the shortest road distance in kilometres between the centre of each postcode and these health facilities. The average distance for all postcodes within each Lower layer Super Output Area (LSOA) level was then estimated and linked to survey responses based on the LSOA in which the respondent lived.	N/A	N/A
Healthcare usage	SANAD II trial	Have you been to a hospital casualty/A&E/ urgent care department in the past 12 months?	1=Yes; 2=No<br>If yes, please specify total number	N/A

For peer review only

STROBE Statement—checklist of items that should be included in reports of observational studies

	Item No	Recommendation	Page in manuscript
**Title and abstract**	1	(*a*) Indicate the study's design with a commonly used term in the title or the abstract	
		(*b*) Provide in the abstract an informative and balanced summary of what was done and what was found	Abstract
**Introduction**			
Background/rationale	2	Explain the scientific background and rationale for the investigation being reported	3
Objectives	3	State specific objectives, including any prespecified hypotheses	4
**Methods**			
Study design	4	Present key elements of study design early in the paper	4
Setting	5	Describe the setting, locations, and relevant dates, including periods of recruitment, exposure, follow-up, and data collection	4
Participants	6	(*a*) *Cohort study*—Give the eligibility criteria, and the sources and methods of selection of participants. Describe methods of follow-up<br>*Case-control study*—Give the eligibility criteria, and the sources and methods of case ascertainment and control selection. Give the rationale for the choice of cases and controls<br>*Cross-sectional study*—Give the eligibility criteria, and the sources and methods of selection of participants	4
		(*b*) *Cohort study*—For matched studies, give matching criteria and number of exposed and unexposed<br>*Case-control study*—For matched studies, give matching criteria and the number of controls per case	
Variables	7	Clearly define all outcomes, exposures, predictors, potential confounders, and effect modifiers. Give diagnostic criteria, if applicable	5
Data sources/ measurement	8*	For each variable of interest, give sources of data and details of methods of assessment (measurement). Describe comparability of assessment methods if there is more than one group	*5*
Bias	9	Describe any efforts to address potential sources of bias	
Study size	10	Explain how the study size was arrived at	5
Quantitative variables	11	Explain how quantitative variables were handled in the analyses. If applicable, describe which groupings were chosen and why	5
Statistical methods	12	(*a*) Describe all statistical methods, including those used to control for confounding	5
		(*b*) Describe any methods used to examine subgroups and interactions	
		(*c*) Explain how missing data were addressed	
		(*d*) *Cohort study*—If applicable, explain how loss to follow-up was addressed<br>*Case-control study*—If applicable, explain how matching of cases and controls was addressed<br>*Cross-sectional study*—If applicable, describe analytical methods taking account of sampling strategy	

For peer review only

(*e*) Describe any sensitivity analyses

Continued on next page

For peer review only

For peer review only - http://bmjopen.bmj.com/site/about/guidelines.xhtml

Results			Page in the manuscript
Participants	13*	(a) Report numbers of individuals at each stage of study—eg numbers potentially eligible, examined for eligibility, confirmed eligible, included in the study, completing follow-up, and analysed	6
		(b) Give reasons for non-participation at each stage	
		(c) Consider use of a flow diagram	
Descriptive data	14*	(a) Give characteristics of study participants (eg demographic, clinical, social) and information on exposures and potential confounders	6
		(b) Indicate number of participants with missing data for each variable of interest	
		(c) *Cohort study*—Summarise follow-up time (eg, average and total amount)	
Outcome data	15*	*Cohort study*—Report numbers of outcome events or summary measures over time	
		Case-control study—Report numbers in each exposure category, or summary measures of exposure	
		Cross-sectional study—Report numbers of outcome events or summary measures	*6*
Main results	16	(*a*) Give unadjusted estimates and, if applicable, confounder-adjusted estimates and their precision (eg, 95% confidence interval). Make clear which confounders were adjusted for and why they were included	6
		(*b*) Report category boundaries when continuous variables were categorized	
		(*c*) If relevant, consider translating estimates of relative risk into absolute risk for a meaningful time period	
Other analyses	17	Report other analyses done—eg analyses of subgroups and interactions, and sensitivity analyses	
**Discussion**			
Key results	18	Summarise key results with reference to study objectives	7-9
Limitations	19	Discuss limitations of the study, taking into account sources of potential bias or imprecision. Discuss both direction and magnitude of any potential bias	9
Interpretation	20	Give a cautious overall interpretation of results considering objectives, limitations, multiplicity of analyses, results from similar studies, and other relevant evidence	7-9
Generalisability	21	Discuss the generalisability (external validity) of the study results	7-10
**Other information**			
Funding	22	Give the source of funding and the role of the funders for the present study and, if applicable, for the original study on which the present article is based	11

*Give information separately for cases and controls in case-control studies and, if applicable, for exposed and unexposed groups in cohort and cross-sectional studies.

For peer review only

For peer review only - http://bmjopen.bmj.com/site/about/guidelines.xhtml

Note: An Explanation and Elaboration article discusses each checklist item and gives methodological background and published examples of transparent reporting. The STROBE checklist is best used in conjunction with this article (freely available on the Web sites of PLoS Medicine at http://www.plosmedicine.org/, Annals of Internal Medicine at http://www.annals.org/, and Epidemiology at http://www.epidem.com/). Information on the STROBE Initiative is available at www.strobe-statement.org.

For peer review only

What are the social predictors of Accident & Emergency attendance in disadvantaged neighbourhoods? Results from a cross-sectional household health survey in the North West of England

Journal:	*BMJ Open*
Manuscript ID	bmjopen-2018-022820.R3
Article Type:	Research
Date Submitted by the Author:	18-Oct-2018
Complete List of Authors:	Giebel, Clarissa; University of Liverpool, Institute of Psychology, Health & Society; CLAHRC NWC<br>McIntyre, Jason; University of Liverpool, Institute of Psychology, Health & Society<br>Daras, Konstantinos ; University of Liverpool, Geographic Data Sciences Lab<br>Gabbay, Mark; University of Liverpool, Institute of Psychology, Health & Society; CLAHRC NWC<br>Downing, Jennifer; University of Liverpool, Department of Molecular and Clinical Pharmacology<br>Pirmohamed, Munir; The University of Liverpool, Department of Pharmacology; CLAHRC NWC<br>Walker, Fran; CLAHRC NWC<br>Sawicki, Wojciech; Royal Liverpool and Broadgreen University Hospitals NHS Trust<br>Alfirevic, Ana; University of Liverpool, Institute of Translational Medicine; CLAHRC NWC<br>Barr, Ben ; University of Liverpool, Institute of Psychology, Health & Society; CLAHRC NWC
**Primary Subject Heading**:	Public health
Secondary Subject Heading:	Health services research
Keywords:	accident and emergency, service access, Depression & mood disorders < PSYCHIATRY, deprivation, housing quality

SCHOLARONE™
Manuscripts

What are the social predictors of Accident & Emergency attendance in disadvantaged neighbourhoods? Results from a cross-sectional household health survey in the North West of England

Clarissa Giebel* *Research Manager*[1,2], Jason C. McIntyre* *Lecturer*[3], Konstantinos Daras *Data Scientist*[4], Mark Gabbay *Professor*[1,2], Jennifer Downing *Research Manager*[2,5], Munir Pirmohamed *Professor*[2,4], Fran Walker *Public Advisor*[2], Wojciech Sawicki *Consultant*[6], Ana Alfirevic* *Reader*[2,5], Ben Barr* *Senior Clinical Lecturer*[1,2]

[1]Institute of Psychology, Health and Society, University of Liverpool, England; [2]NIHR Collaboration for Leadership in Applied Health Research and Care North West Coast, England; [3]School of Natural Sciences and Psychology, Liverpool John Moore's University; [4]Geographic Data Science Lab, University of Liverpool, England; [5]Institute of Translational Medicine, University of Liverpool, England; [6]Royal Liverpool and Broadgreen University Hospitals NHS Trust, Liverpool, England
*joint first authors, and joint senior authors

Correspondence to: Clarissa Giebel, Waterhouse Building B Block, Institute of Psychology, Health & Society, University of Liverpool, Liverpool L69 3GL, England. Email: Clarissa.giebel@liverpool.ac.uk; Tel.: +44 (0) 151 795 5390

For peer review only

Total word Count: 2,341
Keywords: Accident and emergency attendance; service access; depression; deprivation; housing quality

Abstract

Objectives The aim of this study was to identify the most important determinants of Accident & Emergency (A&E) attendance in disadvantaged areas.

Design, setting, and participants A total of 3,510 residents from 20 disadvantaged neighbourhoods in the North West Coast area in England completed a comprehensive public health survey.

Main outcome measures Participants were asked to complete general background information, as well as information about their physical health, mental health, lifestyle, social issues, housing and environment, work and finances, and health care service usage. Only one resident per household could take part in the survey. Poisson regression analysis was employed to assess the predictors of A&E attendance frequency in the previous 12 months.

Results 31.6 percent of the sample reported having attended A&E in the previous 12 months, ranging from 1 to 95 visits. Controlling for demographic and health factors, not being in employment and living in poor quality housing increased the likelihood of attending an A&E service. Service access was also found to be predictive of A&E attendance insofar as there was an additional 18 fewer A&E attendances per 100 population for each kilometre closer a person lived to a General Practitioner (GP) practice, and 3 fewer attendances per 100 population for each kilometre further a person lived from an A&E department.

Conclusions This is one of the first surveys to explore a comprehensive set of socio-economic factors as well as proximity to both GP and A&E services as predictors of A&E attendance in disadvantaged areas. Findings from this study suggest the need to address

For peer review only - http://bmjopen.bmj.com/site/about/guidelines.xhtml

both socioeconomic issues, such as employment and housing quality, as well as structural issues, such as public transport and access to primary care, to reduce the current burden on A&E departments.

Strengths and limitations of this study

- This survey is novel insofar as it examines a comprehensive set of factors linked to A&E attendance, including physical health, mental health, service access and socio-economic factors.
- Participants were recruited from a wide geographical area of disadvantaged neighbourhoods, which strengthens the representativeness of the survey findings.
- Households were approached at different times during the day to produce a less biased sample.
- Some of the most disadvantaged people may not be captured in the survey due to not having a fixed address.

For peer review only

For peer review only - http://bmjopen.bmj.com/site/about/guidelines.xhtml

INTRODUCTION

Accident and emergency (A&E) attendance rates are rapidly increasing in the UK and are particularly high in disadvantaged areas. This is leading to unsustainable pressure on the NHS, with 20.5 million attendances reported in England from April 2015 to March 2016, compared to 19.5 million the previous year [1]. According to a King's Fund report, unnecessary A&E attendances could be reduced by between eight and 18% if patients attended a primary care service for their health concerns instead [2]. The predictors of A&E attendance are complex and involve multiple components. Whilst we know that A&E attendances are high in disadvantaged areas [3], the specific reasons for this are less clear. The prevalence of health problems is likely to be important; however, other factors may also play a role. Housing problems and employment status, for example, have been associated with high levels of A&E attendance, as have loneliness and low levels of social support [4,5]. Health service factors, including proximity to A&E and poor access to primary care, have also been linked to higher A&E attendance rates in past research [3, 6-11].

Previous studies on predictors of A&E attendance have been limited because they have not assessed the independent influence of the full range of potential risk factors, including morbidity, socio-economic factors, social support and proximity to A&E and primary care. In a recent study by Hull and colleagues [12], primary and secondary care data were linked with A&E attendance data in London. The data showed that multimorbidity, smoking, increased number of GP attendances, shorter distance to A&E, and age were all linked to visits to an A&E department. By comparing the most

For peer review only - http://bmjopen.bmj.com/site/about/guidelines.xhtml

disadvantaged *versus* the least disadvantaged quintile, Hull and colleagues [12] reported a 52% higher A&E attendance in the most disadvantaged population sample.

The aim of this study was therefore to use data from a household health survey conducted in disadvantaged neighbourhoods in the North West of England to identify the most important determinants of A&E attendance in disadvantaged populations to inform actions that could reduce demand for A&E services.

METHODS

Participants

The Northwest Coast area of England contains some of the most disadvantaged neighbourhoods in the country [13]. The Household Health Survey (HHS) of the National Institute for Health Research Collaboration for Leadership in Applied Health Research and Care in the North West Coast (NIHR CLAHRC NWC) was conducted between August 2015 and January 2016 to identify individual and neighbourhood level factors that contribute to inequalities in physical and mental health and to provide a baseline for evaluating neighbourhood level public heath interventions.

Twenty disadvantaged neighbourhoods were initially selected by participating local authorities. Each of the 10 Local Authorities that was part of the NIHR CLAHRC NWC partnership were asked to select a neighbourhood that would be involved in a number of public health initiatives and a comparator area with similar characteristics. The local authorities were asked to select these neighbourhood based on the following criteria: they (1) had a population between 5,000 and 10,000 residents; (2) had a deprivation index [13] in the bottom 15% of the national average and 3) had a coherent or shared sense of

For peer review only

identity among residents. In addition the intervention areas needed to have a local community organisation that could support the implementation of the public health interventions. The selection of these neighbourhoods was at the discretion of the participating local authorities. The public health initiatives commenced in 10 of the neighbourhoods after the survey data was collected. An additional sample (N=809) was also collected from 8 less disadvantaged neighbourhoods, however data from this sample was not used in this study.

Prior to the survey, ethical approval was obtained from the University of Liverpool (Ref: RETH000836). The survey fieldwork was conducted by BMG research and a pilot survey was completed with 36 residents from the disadvantaged neighbourhoods prior to the full data collection, which resulted in minor changes to survey documents. A random sample of households was then drawn from each of the disadvantaged neighbourhoods and one randomly selected respondent completed a face-to-face interview in each household (N=3,510). The survey combined information on a wide range of socioeconomic factors, mental and physical morbidities, and healthcare utilisation. Full details of the survey methodology are published elsewhere [14].

Variables

Our outcome variable was defined as the number of times respondents reported attending an A&E department over the previous twelve months. Measures of socioeconomic conditions included education (no qualifications, professional or vocational certificate, Degree or higher), employment (working/not working), financial hardship (doing well/getting by, struggling), change in financial circumstances (getting better or

For peer review only - http://bmjopen.bmj.com/site/about/guidelines.xhtml

unchanged/worse than 12 months ago), housing quality (no problems / problems with cold, damp, or mould). Physical health was assessed with the four physical health dimensions of the EuroQuol five-dimension scale (EQ-5D) [15] for mobility, self-care, engagement in usual activities and pain (no problems/ some or severe problems). Mental health was assessed using a series of validated instruments. Depression was measured using the nine-item Patient Health Questionnaire [16]. Anxiety was measured using the seven-item Generalized Anxiety Disorder scale [17]. Paranoia was measured using the persecution subscale of the persecution and deservedness scale (PaDS) for symptoms of paranoia [18]. Practical social support and social contact were assessed based on the level of agreement with the statements "If I needed help, there are people who would be there for me" and "If I wanted company or to socialize, there are people I can call on". Response options for both items ranged from 1 = *definitely disagree* to 4 = *definitely agree*. (Further information on each of the survey variables is given in Web Appendix 1).

The proximity to A&E departments and GP practices was estimated using the Routino open source tool[1] to calculate the shortest road distance between the centre of each postcode and these health facilities. The average distance for all postcodes within each Lower layer Super Output Area (LSOA) level[2] was then estimated and linked to survey responses based on the LSOA in which the respondent lived.

Public involvement

Members of the public have been involved throughout the design and implementation of the survey, in addition to external partners and representatives from local authorities. This

[1] https://www.routino.org/uk/

[2] A Lower layer Super Output Area (LSOA) is a geographical area in the UK which is based on postcodes and clusters together groups of the population.

For peer review only - http://bmjopen.bmj.com/site/about/guidelines.xhtml

For peer review only

involvement included attending research and design meetings, providing feedback on study documents, as well as helping with the dissemination of findings. Public advisors have been recruited from the sampled disadvantaged areas and receive monetary reimbursement for their involvement.

Data analysis

Data were analysed using Stata version 14. Because the A&E attendance variable comprised highly skewed (*S-W* = .39, p < .001) count data, we constructed a Poisson regression model. Standard errors were adjusted to account for the multi-stage nature of the survey sampling using the *svyset* commands in Stata [19] and the analysis was weighted for non-response. This model provided estimates of the Rate Ratio (RR) of A&E attendance associated with each variable, while holding all other variables constant in the model. We then used this model to calculate the Absolute Rate Differences (ARD) as the marginal effect of each of the variables whilst holding the other variables at their means. Demographic, socio-economic, health condition, disability, symptom, and healthcare access variables were entered into the model.

RESULTS

1,109 of the 3,510 survey respondents (31.6%) reported attending A&E at least once in the past 12 months. Among those, the number of attendances ranged from 1 to 95 (M=2.70, SD=4.90) and the overall rate of A&E attendance was 75 attendances per 100 population (95% CI 64, 87).

For peer review only - http://bmjopen.bmj.com/site/about/guidelines.xhtml

Table 1 shows the results of the Poisson regression model as well as descriptive statistics for the independent variables. Rate ratios (RR) and Absolute rate Differences (ARD) are reported alongside confidence intervals. Age was a significant demographic predictor of A&E attendances. After adjusting for other variables in the model the A&E attendance rate was three times higher amongst 18-24 year olds compared to people over 64 years of age (RR=2.72, 95%CI 1.33-5.53). In absolute terms, this was equivalent to an additional 60 attendances per 100 18-24 year olds compared to over 65 year olds, other factors being held constant (ARD = 60 per 100, 95% CI 10, 110). The health and disability variables predictive of A&E attendance included having a health condition as opposed to no health condition (RR=1.78, 95%CI 1.19-2.66, ARD = 28 per 100, 95%CI 10, 45), and having problems with self-care such as washing and dressing (RR=2.63, 95%CI 1.82-3.79; ARD = 71 per 100, 95%CI 34, 109). People with multiple morbidities were not more likely to attend A&E than people with only one condition. Of the socio-economic and environmental factors significantly associated with A&E attendance, being out of work increased risk of A&E attendance by 38% (RR=1.38, 95%CI 1.08-1.78; ARD = 16 per 100, 95%CI 4, 27) and living in a house with problems with cold, mould or damp increased risk of A&E attendance by 34% (RR=1.34, 95%CI 1.01 to 1.76; ARD = .14 per 100, 95%CI 1, 27). Surprisingly in this sample of people living in relatively disadvantaged neighbourhoods, higher levels of education were associated with higher risk of A&E attendance when controlling for other factors. Levels of social support and social contact did not predict A&E attendance.

Both healthcare access variables were significantly related to A&E attendance. Specifically, living further from an A&E department reduced the likelihood of attending an

For peer review only - http://bmjopen.bmj.com/site/about/guidelines.xhtml

A&E service by seven percent per kilometre (RR = 0.93, 95%CI 0.89,0.97), and living further from a GP increased the likelihood of attending A&E by 46 percent per kilometre, after controlling for health status, socioeconomic and demographic factors (RR = 1.46, 95%CI 1.12, 1.90). Absolute Rate Differences indicated that for each kilometer closer the average person lived to an A&E department, they had 3 fewer A&E attendances per 100 population in the previous 12 months (ARD = -3, 95%CI -5, -2). Conversely, there were 18 more A&E attendances per 100 population for each kilometre further the average person lived from a GP practice (ARD = 18, 95%CI 6, 30).

For peer review only

DISCUSSION

This is one of the first surveys to collect data on socio-economic background, mental and physical health and health care utilisation, and to combine these factors with distance to health care services. Spanning a wide geographical area in one of the most disadvantaged areas of England [13], findings from the survey identify several key risk factors of A&E attendance, including young age, depression, high education, non-employment, poor housing, as well as longer distance from a GP and shorter distance to an A&E service.

Considering the increase in A&E attendance across England [1], particularly in disadvantaged areas [3], it is important to understand the reasons behind this rise and to design strategies to reduce health inequalities. A&E attendance was significantly higher for 18 to 24 year olds, when controlling for other factors, which has been found in previous work [20], and may be due to heightened rates of accidents, trauma and alcohol-related attendances at A&E [21], or higher use of A&E for primary care reasons in this age group. A limitation of our study is that we did not collect any information on the reasons for

For peer review only - http://bmjopen.bmj.com/site/about/guidelines.xhtml

A&E attendance. Furthermore, A&E attendance has to be distinguished from admission to hospital, which we know is more prevalent in the elderly with long-term conditions [22].

Higher levels of education were linked to increased risks of attending A&E services in our disadvantaged sample. Considering that people from a low socioeconomic background are more likely to attend A&E services, as shown in this survey and supported by other research [23-27], it is perhaps surprising to find those with a professional or degree level education to be up to twice as likely to access A&E when holding all other factors constant. It should be noted however that this is only the case when adjusting for other health and socioeconomic factors – the crude risk ratios for education were all not significantly different from 1. So it appears in our sample that where people with higher levels of education are living in disadvantaged areas and have similar levels of health problems as the rest of the population and are living in similar social circumstances they are more likely to attend A&E than there less educated peers. One possible reason for this may be that higher educated residents are more likely to recognise specific symptoms and become pro-active by seeking medical help. It is also plausible that people with higher qualifications have ended up living in disadvantaged neighbourhood, due to their poor health and this leads to increased A&E attendances. Indeed, depression as well as problems with self-care were independently associated with more A&E visits, supporting previous evidence of increased emergency attendances among people with mental health problems who are more likely to be "frequent users" [6, 28-29]. Thus, interventions that address mental health problems, particularly depression, and that provide greater support to people who have problems with self-care may be linked to concomitant declines in A&E attendance. However, the relationship between depression and A&E attendance should be

For peer review only - http://bmjopen.bmj.com/site/about/guidelines.xhtml

interpreted with some caution because the rate ratio was found to be significant, while the absolute risk difference was not significant.

Being out of work and living in poor housing were important predictors of increased A&E attendance, above and beyond the effect of health status. This could be because these factors exacerbate existing conditions. Indeed, unemployment has been linked to higher A&E attendances for asthma [30], whilst poor housing quality has been linked to more asthma-related symptoms [31] and worse mental health [32]. Controlling for these socioeconomic factors, the Index of Multiple Deprivation (IMD) was not independently associated with A&E attendance within this sample of disadvantaged areas.[3] This indicates that much of the high level of A&E attendance found by others in disadvantaged areas [3] is possibly explained by differences in the prevalence of health conditions, high unemployment and poor housing. This lack of an effect related to deprivation may also be related to the limited variation in deprivation between the relatively deprived areas in our sample. In contrast, social support was not found to be linked to A&E attendance. Whilst a strong social network offering practical and emotional support is important for well-being [33], this did not affect attendance of A&E services in our study. It may be the case that participants may have genuinely needed to access healthcare services for their physical and mental health needs which could not be supported otherwise. Therefore, interventions that improve housing conditions and promote employment in disadvantaged communities should be integral to strategies to reduce demand for A&E services.

[3] To check for multicollinearity we regressed each individual socioeconomic variable onto IMD, using dummy coding for categorical predictors. All standardised coefficients were < .26, suggesting that IMD was not collinear with the other socioeconomic measures.

For peer review only - http://bmjopen.bmj.com/site/about/guidelines.xhtml

We found that longer distance from GP practices and shorter distance to A&E departments were significant predictors of A&E attendance. The findings are consistent with previous work on A&E proximity [8,9;12]; however, we additionally identified GP practice proximity as a unique determinant of A&E attendance. The results indicate that primary care access predicts A&E attendance over and above the effects of health status, socioeconomic status, and A&E access. The current trend of consolidating GP practices in fewer, larger health centres may therefore be contributing to increases in A&E attendances. This trend may also be linked to ease of access, in that patients may be unable to get a GP appointment and therefore attend an A&E service instead. Future approaches should be cognisant of primary care service placement and access. Reduced demand for A&E may be achieved via improved public transport links that consider primary care locations, or via strategically positioning primary care services in areas with high A&E attendance rates. It would be of interest in future research to examine whether factors such as transport links or appointment availability independently predict A&E attendance or, in the case of public transport links, explain the relationship between distance to services and service usage.

One recently conducted retrospective study has also explored the socioeconomic predictors of A&E attendance [12], showing that A&E attendance increased with increasing numbers of health conditions, in addition to risk of A&E attendance increase with smoking, GP attendance, being housebound, and age. However, the study differed in several aspects from the household health survey. On the one hand, the study by Hull and colleagues [12] did not measure the effects of proximity to GP services. In addition, our household health survey contains a rich set of mental health, health related quality of life

For peer review only - http://bmjopen.bmj.com/site/about/guidelines.xhtml

and socioeconomic variables, whereas the London-based assessment of primary and secondary care data only employs the IMD as a measure of social deprivation, having no information on for example housing quality, whilst health measures were limited to numbers of conditions. In our study A&E attendance did not increase with increasing numbers of health conditions. One plausible explanation for this effect is that the number of health conditions a person has is not in itself a risk for A&E attendance – but this is just a marker of people with poorer mental health and health related quality of life. Further research is required to confirm whether this explains the divergent findings.

The findings from this survey should be considered in light of a few limitations. The cross-sectional nature of the survey only allowed investigation of the predictors of A&E attendance at one point in time. Moreover, this survey was based on self-reports, and thus participants had to remember whether they had attended an A&E department, and how often, in the past 12 months. For this reason, there could potentially have been some recall bias in the number of A&E attendances. This could be avoided by referring to Hospital Episodes Statistics data from the National Health Service. The survey also purposefully recruited residents from some of the most disadvantaged areas within England and the Northwest Coast, and selected only neighbourhoods that met criteria that included methodological (e.g., large population and shared neighbourhood identity) and practical (e.g., infrastructure for interventions) constraints. Thus, the findings may not generalise to other areas that did not meet these criteria. Examining such a wide range of determinants meant striking a balance between including multiple determinants and examining those determinants in detail. Because of this, examining more nuanced research questions, such as the role of different types of health conditions on A&E

For peer review only - http://bmjopen.bmj.com/site/about/guidelines.xhtml

attendance, was not possible. Lastly, people without a fixed address who may be living in extremely disadvantaged circumstances were probably not captured by our data.

Conclusions

We conducted a cross-sectional analysis of A&E attendance risk factors in a large sample of UK residents in disadvantaged neighbourhoods. To our knowledge, this is the most comprehensive examination of diverse socioeconomic determinants of A&E attendance, and the first to examine GP proximity alongside A&E proximity. Housing quality, employment status, self-care difficulties, depression, and proximity to both primary and emergency services were identified as the most important determinants of A&E attendance. The findings highlight the need for multi-pronged approaches to reduce A&E attendances that address socioeconomic inequalities, such as employment and housing quality, but also structural issues such as access to primary care. Key policy suggestions likely to reduce A&E attendance based on these findings include: strategic placement of — and better transport links to — primary care services, better housing conditions, increased employment and improved support for self-care and public mental health. Improving housing conditions has shown short-term benefits on the health of residents, without having evaluated A&E attendance rates [31; 34]. To date, findings on the effect of improving primary care access interventions on reducing A&E attendance appear inconclusive [35], suggesting the need for further implementation strategies to improve primary care access. Strategies to reduce strain on health services need to address the social and economic factors that underpin demand for healthcare and enhance access to primary care.

For peer review only - http://bmjopen.bmj.com/site/about/guidelines.xhtml

Funding

This work was supported by the National Institute for Health Research, Collaboration for Leadership and Health Research and Care North West Coast (NIHR CLAHRC NWC). The views expressed are those of the author(s) and not necessarily those of the NHS, the NIHR or the Department of Health.

Competing interests

We have read and understood BMJ policy on declaration of interests and declare the following: All authors have completed the ICMJE uniform disclosure form. The authors have no financial relationships with any organisations that might have an interest in the submitted work in the previous three years, no other relationships or activities that could appear to have influenced the submitted work.

Author Contributions

CG and JMI jointly wrote the article, and JMI and BB performed statistical analysis. KD, MP, MG, JD, FW, WS, AA, and BB edited and provided feedback on drafts of the manuscript and approved the final version.

Research ethics

The research was approved by the University of Liverpool Committee on Research Ethics (Ref: RETH00836). Participants provided informed consent before completing the study.

For peer review only

For peer review only - http://bmjopen.bmj.com/site/about/guidelines.xhtml

Data sharing statement

The dataset from the North West Coast Household Health Survey will be made publicly available after an embargo period.

For peer review only

For peer review only - http://bmjopen.bmj.com/site/about/guidelines.xhtml

References

[1] NHS Digital, 2017. Hospital Accident and Emergency Activity 2015-2016. Health and Social Care Information Centre, London: NHS Digital.

[2] Tian Y. 2012. Data briefing: emergency hospital admissions for ambulatory care-sensitive conditions. King's Fund report. https://www.kingsfund.org.uk/sites/default/files/field/field_publication_file/data-briefing-emergency-hospital-admissions-for-ambulatory-care-sensitive-conditions-apr-2012.pdf (accessed 28 Sep 2017).

[3] Scantlebury R, Rowlands G, Durbaba S, Schofield P, Sidhu K, Ashworth M. Socioeconomic deprivation and accident and emergency attendances: cross-sectional analysis of general practice in England. Br J Gen Pract 2015;65(639):e649-e654.

[4] Geller J, Janson P, McGovern E, Valdini A. Loneliness as a Predictor of Hospital Emergency Department Use. The Journal of Family Practice 1999;48(10): 801–4.

[5] Molloy GJ, McGee HM, O'Neill D, Conroy RM. Loneliness and Emergency and Planned Hospitalizations in a Community Sample of Older Adults. *Journal of the American Geriatrics Society* 2010;58(8): 1538–41.

[6] Daniels J, Osborn M, Davis C. Better safe than sorry? Frequent attendance in a hospital emergency department: an exploratory study. Br J Pain 2017; doi:10.1177/2049463717720635

[7] Hull SA, Jones IR, Moser K. Factors influencing the attendance rate at accident and emergency departments in East London: The contributions of practice organization, population characteristics and distance. J Health Services Research Policy 1997;2(1):6-13.

[8] Paul P, Heng BH, Seow E, Molina J, Tay SY. Predictors of frequent attenders of emergency department at an acute general hospital in Singapore. Emergency Medical Journal 2010;27:805-805.

[9] Rudge GM, Mohammed MA, Fillingham SC, et al. The Combined Influence of Distance and Neighbourhood Deprivation on Emergency Department Attendance in a Large English Population: A Retrospective Database Study. PLOS One 2013; doi: 10.1371/journal.pone.0067943

[10] Takahashi PY, Ryu E, Hathcock MA, et al. A novel housing-based socioeconomic measure predicts hospitalization and multiple chronic conditions in a community population. J Epidemiology Community Health 2016;70(3):286-291.

[11] Tammes P, Morris RW, Brangan E, et al. Exploring the relationship between general practice characteristics, and attendance at walk-in centres, minor injuries units and EDs in England 2012/2013: a cross-sectional study. BMJ 2017;33:702-708.

[12] Hull SA, Homer K, Boomla K, Robson J, Ashworth M. Population and patient factors affecting emergency department attendance in London: retrospective cohort analysis of linked primary and secondary care records. Br J Gen Pract 2018; doi: 10.3399/bjgp18X694397

[13] Department for Communities and Local Government. The English Indices of Deprivation 2015: technical report. London: 2015.

For peer review only - http://bmjopen.bmj.com/site/about/guidelines.xhtml

[14] McIntyre J, Wickham S, Barr B, Bentall RP. Social Identity and Psychosis: Associations and Psychological Mechanisms. Schizophrenia Bulletin 2017, doi: 10.1093/schbul.sbx110.
[15] Herdman M, Gudex C, Lloyd A, Janssen MF, Kind P, Parkin D, Bonsel G, Badia X. Development and preliminary testing of the new five-level version of EQ-5D (EQ-5D-5L). Quality of Life Research 2011;20(10):1727-1736.
[16] Kroenke K, Spitzer RL, Williams JBW. The PHQ-9 – Validity of a Brief Depression Severity Measure. J Gen Intern Med 2001;16(9):606-613.
[17] Spitzer RL, Kroenke K, Williams JBW, Loewe B. A brief measure for assessing Generalized Anxiety Disorder – The GAD-7. Arch Intern Med 2006;166:1092-1097.
[18] Melo S, Corcoran R, Shryane N, Bentall RP. The persecution and deservedness scale. Psychol Psychother 2009;82:247-260.
[19] StataCorp. 2015. Stata Survey Data Reference Manual. College Station, TX: StataCorp LLC.
[20] Health and Social Care Information Centre. *Hospital Accident and Emergency Activity: 2015-16*. NHS Digital, England: 2017.
[21] Parkinson K, Newbury-Birch D, Phillipson A, et al. Prevalence of alcohol related attendance at an inner city emergency department and its impact: a dual prospective and retrospective cohort study. Emergency Medicine Journal 2016;33:187-193.
[22] King's Fund. *Long-term conditions and multi-morbidity*. Last accessed 15th January 2018: https://www.kingsfund.org.uk/projects/time-think-differently/trends-disease-and-disability-long-term-conditions-multi-morbidity
[23] Krieg C, Hudon C, Chouinard MC, Dufour I. Individual predictors of frequent emergency department use: a scoping review. BMC Health Serv Res 2016; 16:594.
[24] McKetin R, Degenhardt L, Shanahan M, et al. Health service utilization attributable to methamphetamine use in Australia: Patterns, predictors and national impact. Drug Alc Rev 2018;37(2):196-204.
[25] Pines JM, Buford K. Predictors of frequent emergency department utilization in Southeastern Pennsylvania. J Asthma. 2006;43(3):219–23.
[26] Sun BC, Burstin HR, Brennan TA. Predictors and outcomes of frequent emergency department users. Acad Emerg Med. 2003;10(4):320–8.
[27] Zuckerman S, Shen YC. Characteristics of occasional and frequent emergency department users: do insurance coverage and access to care matter? Med Care. 2004;42(2):176–82.
[28] Keene J, Rodriguez J. Are mental health problems associated with use of Accident and Emergency and health-related harm? European J Public Health 2007;17(4):387-393.
[29] Ingram WM, Weston C, Ritchie MD, Larson S. Depression linked to frequent emergency department use in large 10-year retrospective analysis of an integrated health care system. bioRxiv 2017; doi:10.1101/115238
[30] Forbes L, Harvey S, Newson R, et al. Risk Factors for Accident and Emergency (A&E) Attendance for Asthma in Inner City Children. Thorax 2007;62(10):855–60.
[31] Barton A, Basham M, Foy C, Buckingham K, Somerville M. The Watcombe Housing Study: the short term effect of improving housing conditions on the health of residents. Journal of Epidemiology Community Health 2007;61(9):771-777.

For peer review only - http://bmjopen.bmj.com/site/about/guidelines.xhtml

[32] Evans GW, Wells NM, Chan HYE, Saltzman H. Housing quality and mental health. J Consulting Clin Psychol 2000;68(3):526-530.

[33] Gilles I, Clemence A, Courvoisier N, Sanchez S. Social support, social and temporal comparisons protect well-being and health between 45 and 70 years old in four urban communities. Aging Sci 2015;3(1): doi: 10.4172/2329-8847.1000133

[34] Woodfine L, Neal RD, Bruce N, Edwards RT, Linck P, Mullock L, et al. Enhancing ventilation in homes of children with asthma: pragmatic randomized controlled trial. Br J Gen Pract 2011;61(592):e727-e732.

[35] Ismail SA, Gibbons DC, Gnani S. Reducing inappropriate accident and emergency department attendances: a systematic review of primary care service interventions. Br J Gen Pract 2013;63(617):e813-e820.

For peer review only

For peer review only - http://bmjopen.bmj.com/site/about/guidelines.xhtml

Table 1. Determinants of number of A&E attendances over the previous 12 months (n=3,510)

Socioeconomic factors	Adjusted Rate Ratio of A&E attendance	Adjusted Rate Ratio 95% CI	Absolute Risk Difference of A&E attendance	Absolute Risk Difference 95% CI	Unadjusted Rate Ratios (95% CI)	N (%)	M (SD)
**Demographics**							
Age (65+)						858 (24.5)	-
18-24	2.72**	1.33, 5.53	.60*	.10, 1.10	1.15 (.70,1.88)	368 (10.5)	-
25-44	1.43	.95, 2.16	.15	-.01, .31	.83 (.62,1.12)	1226 (34.9)	-
45-64	1.21	.83, 1.77	.07	-.07, .21	1.18 (.81,1.72)	1057 (30.1)	-
Gender (female)	.90	.69, 1.18	-.05	-.18, .08	.89 (.66,1.20)	2029 (57.8)	-
Ethnicity (BME)	.60	.35, 1.04	-.20*	-.37, -.03	.40 (.24,.67)	366 (10.5)	-
**Socioeconomic status**							
Education (No qualifications)						1516 (43.3)	-
Professional/vocational certificate	1.58**	1.17, 2.14	.20**	.07, .33	1.20 (.88, 1.63	1579 (45.1)	-
Degree or higher	2.00*	1.15, 3.48	.35*	.02, .67	1.21 (.71, 2.07)	405 (11.6)	-
Non-employment (Not in paid/self-employment)	1.38*	1.08, 1.78	.16**	.04, .27	2.20 (1.65, 2.93)	2150 (61.3)	-
Index of Multiple Deprivation	.99	.99, 1.00	-.003	-.01, .001	.99 (.99, 1.01)	-	47.20 (16.90)
Financial struggle ("Doing well")						716 (20.4)	-
"Getting by"	1.00	.72, 1.40	<.001	-.16, .16	1.33 (.96, 1.86)	2307 (65.8)	-
"Struggling"	1.22	.75, 1.98	.10	-.16, .36	2.94 (1.78, 4.85)	481 (13.7)	-
Financial situation worse than 12 months ago	.99	.78, 1.26	-.004	-.12, .11	1.49 (1.07, 2.09)	578 (16.6)	-
**Housing quality**							
Problems with condensation/mold/ temperature	1.34*	1.01, 1.76	.14*	.01, .27	1.73 (1.26, 2.38)	1138 (33.7)	-
**Health status**							
Mobility problems (EQ5D)	1.24	.84, 1.83	.11	-.10, .32	3.54 (2.64, 4.76)	930 (73.5)	-
Self-care problems (EQ5D)	2.63***	1.82, 3.79	.71***	.34, 1.09	5.58 (3.85, 8.09)	381 (10.9)	-
Problems engaging in usual activities (EQ5D)	1.43*	1.01, 2.03	.19	-.02, .40	4.17 (3.12, 5.57)	852 (24.3)	-
Pain (EQ5D)	1.24	.90, 1.71	.11	-.05, .27	3.30 (2.50, 4.36)	1325 (37.8)	-
1 condition (base category: no condition)	1.78**	1.19, 2.66	.28**	.10, .45	3.19 (2.38, 4.27)	857 (24.4)	-
>1 condition (base category: 1 condition)	1.02	.67, 1.55	.01	-.19, .21	2.80 (2.08, 3.77)	1256 (35.8)	-

For peer review only

**Mental Health**							
Depression (PHQ-9)	1.36*	1.01, 1.83	.15	-.003, .30	2.02 (1.72, 2.37)	-	1.56 (.68)
Anxiety (GAD-7)	.86	.65, 1.14	-.07	-.21, .06	1.75 (1.47, 2.07)	-	1.54 (.75)
Paranoia (PaDS-5)	1.04	.89, 1.21	.02	-.06, .09	1.41 (1.21, 1.65)	-	2.01 (.89)
**Social support**							
Practical support	.93	.47, 1.85	-.03	-.36, .29	.69 (.30, 1.59)	3302 (94.3)	-
Social contact	1.08	.70, 1.70	.04	-.17, .25	.64 (.31, 1.31)	3286 (94.0)	-
**Healthcare access**							
Distance to GP (km)	1.46**	1.12, 1.90	.18**	.06, .30	1.21 (.96, 1.53)		.99 (.66)
Distance to A&E (km)	.93***	.89, .97	-.03***	-.05, -.02	.95 (.91, .99)		7.12(4.21)

* $p < .05$, ** $p < .01$, *** $p < .001$

NOTE: Risk Ratios and Absolute Risk Differences are adjusted for all other variables in the model. BME= Black and Minority Ethnic; EQ5D= Euroqol 5 dimensions; a measure of generic health status; GAD-7=Generalised Anxiety Disorder Assessment; PHQ-9=Patient Health Questionnaire; PaDS-5= Persecution and Deservedness Scale

For peer review only

For peer review only - http://bmjopen.bmj.com/site/about/guidelines.xhtml

Appendix 1: Table of Measures

Variable	Source	Description/Example	Original scoring	Recoding for analysis
Age	Office for National Statistics	N/A	1 = Under 16 years<br>2 = 16-17 years<br>3 = 18-24 years<br>4 = 25-34 years<br>5 = 35-44 years<br>6 = 45-54 years<br>7 = 55-64 years<br>8 = 65-74 years<br>9 = 75+ years	1 = 18-24 years<br>2 = 25-44 years<br>3 = 45-64 years<br>4 = 65+ years
Sex	Office for National Statistics	N/A	1 = Male<br>2 = Female<br>3 = Other	1 = Male<br>2 = Female
Ethnicity	Office for National Statistics	N/A	1 = English / Welsh / Scottish / Northern Irish / British 2 = Irish<br>3 = Gypsy or Irish Traveller<br>4 = Any other White background, *please specify*<br>5 = White and Black Caribbean<br>6 = White and Black African<br>7 = White and Asian<br>8 = Any other Mixed / Multiple ethnic background, *please specify* 9 = Indian<br>10 = Pakistani<br>11 = Bangladeshi<br>12 = Chinese<br>13 = Any other Asian background, *please specify*<br>14 = African<br>15 = Caribbean<br>16 = Any other Black / African / Caribbean background, *please specify*<br>17 = Arab<br>95 = Any other group, please specify	0 = White<br>1 = BME
Non-	Office for National	N/A	1 = Going to school or college full time (including on vacation)	0 = employed

For peer review only

employment	Statistics		2 = In paid employment or self employed (or temporarily away)<br>3 = On a Government scheme for employment training<br>4 = Doing unpaid work for a business that you own, or that a relative owns<br>5 = Waiting to take up paid work already obtained<br>6 = Looking for paid work or a Government training scheme 7 = Intending to look for work but prevented by temporary sickness or injury<br>8 = Permanently unable to work because of long-term sickness or disability 9 = Retired from paid work<br>10 = Looking after the home or family<br>95 = Doing something else, specify	1 = not employed
Education	Office for National Statistics	Do you have any educational qualifications for which you received a certificate?<br><br>Do you have any professional, vocational or other workrelated qualifications for which you received a certificate?<br><br>What is your highest qualification?	1 = Yes, 2 = No<br><br>1 = Yes, 2 = No<br><br>1 = At degree level or above, 2 = Another kind of qualification	1 = No qualifications 2 = Professional or vocational qualification 3 = Degree or higher
Housing quality	English Housing Survey	During the winter months, does condensation form on the windows or walls of any room in your home apart from the bathrooms or toilets?<br><br>During the winter months, are there patches of mould or fungus in any room in your home, apart from bathrooms or toilets?	1 = Yes, 2 = No, 3 = Spontaneous: don't know<br><br>1 = Yes, 2 = No, 3 = Spontaneous: don't know	0 = No problems<br>1 = One problem<br>2 = Two problems 3 = Problems with all three issues

For peer review only

		During the cold winter weather, can you normally keep comfortably warm in your living room?	1 = Yes, 2 = No, 3 = Spontaneous: don't know	
Financial struggle	Wealth and Assets Survey	How well would you say your household is managing financially these days?	1 = Doing well<br>2 = Getting by<br>3 = Struggling	N/A
Financial Situation	Wealth and Assets Survey	Would you say your household is better off or worse off financially than you were a year ago?	1 = Better off<br>2 = About the same 3 = Worse off<br>4 = Don't know	1 = Better off<br>2 = About the same<br>3 = Worse off
Index of Multiple Deprivation (IMD)	Office of National Statistics	Measure of deprivation at level of Lower Level Super Output Area (LSOA) across seven domains: Income, Employment, Education, Health and Disability, Crime, Housing, ad Living Environment.	N/A	N/A
Physical Health	EuroQual Five Dimensional Scale (EQ-5D)	Standardised instrument that assesses problems with mobility, self-care, engagement in usual activities, and pain.	1 = No problems<br>2 = Some Problems/Moderate problems<br>3 = Extreme problems/Unable	0 = No problems 1 = Some/Severe problems
Multimorbidity	Psychiatric Morbidity Survey	Have you ever had any of [these health conditions] over the past 12 months?	1 = Cancer<br>2 = Diabetes<br>3 = Epilepsy/fits<br>4 = Migraine or other frequent headaches<br>5 = Dementia or Alzheimer's disease<br>6 = Any mental health issue<br>7 = Cataracts / eyesight problems (even if corrected with glasses or contacts)<br>8 = Ear/hearing problems (even if corrected with a hearing aid)<br>9 = Stroke	*At least one condition*<br><br>0 = No conditions<br>1 = One condition<br><br>*Multimorbidity*<br><br>0 = One condition 1 = More than one condition

For peer review only

			10 = Heart attack/angina<br>11 = High blood pressure<br>12 = Bronchitis/emphysema<br>13 = Asthma<br>14 = Allergies<br>15 = Stomach ulcer or other digestive problems<br>16 = Liver problems<br>17 = Bowel/colon problems<br>18 = Bladder problems/incontinences<br>19 = Arthritis<br>20 = Bone, back joint or muscle problems<br>21 = Gout<br>22 = Skin problems<br>95 = Other, please specify<br>96 = None of these	
Depression	Patient Health Questionnaire (PHQ-9)	Assesses how often participants had been bothered by pro problems such as "Feeling down, depressed, or hopeless" over the past two weeks.	1 = Not at all<br>2 = Several days<br>3 = More than half the days<br>4 = Nearly every day	N/A
Anxiety	Generalised Anxiety Disorder Questionnaire (GAD-7)	Assesses frequency of events such as "Being so restless that it is hard to sit still" over the past two weeks.	1 = Not at all<br>2 = Several days<br>3 = More than half the days<br>4 = Nearly every day	N/A
Paranoia	Five-item Persecution and Deservedness Scale (PaDS-5)	Assesses the extent to which people are suspicious of others' intentions. Participants rate their level of agreement with statements such as "I'm often suspicious of other people's intentions towards me."	1 = Strongly disagree<br>2 = Disagree<br>3 = Neither agree or disagree<br>4 = Agree<br>5 = Strongly agree	N/A
Social Support	Community Life Survey	Assesses the extent to which participants agree they receive *practical support* ("If I	1 = Definitely agree<br>2 = Tend to agree<br>3 = Tend to disagree	0 = Disagree<br>1 = Agree

For peer review only

		needed help, there are people who would be there for me") and *social contact* (If I wanted company or to socialise, there are people I can call on")	4 = Definitely disagree<br>5 = Don't know	
Healthcare Access	N/A	The *proximity to A&E departments* and *proximity to GP practices* was estimated using the Routino open source tool (Routino.org.uk) to calculate the shortest road distance in kilometres between the centre of each postcode and these health facilities. The average distance for all postcodes within each Lower layer Super Output Area (LSOA) level was then estimated and linked to survey responses based on the LSOA in which the respondent lived.	N/A	N/A
Healthcare usage	SANAD II trial	Have you been to a hospital casualty/A&E/ urgent care department in the past 12 months?	1=Yes; 2=No<br>If yes, please specify total number	N/A

For peer review only

STROBE Statement—checklist of items that should be included in reports of observational studies

	Item No	Recommendation	Page in manuscript
**Title and abstract**	1	(*a*) Indicate the study's design with a commonly used term in the title or the abstract	
		(*b*) Provide in the abstract an informative and balanced summary of what was done and what was found	Abstract
**Introduction**			
Background/rationale	2	Explain the scientific background and rationale for the investigation being reported	3
Objectives	3	State specific objectives, including any prespecified hypotheses	4
**Methods**			
Study design	4	Present key elements of study design early in the paper	4
Setting	5	Describe the setting, locations, and relevant dates, including periods of recruitment, exposure, follow-up, and data collection	4
Participants	6	(*a*) *Cohort study*—Give the eligibility criteria, and the sources and methods of selection of participants. Describe methods of follow-up<br>*Case-control study*—Give the eligibility criteria, and the sources and methods of case ascertainment and control selection. Give the rationale for the choice of cases and controls<br>*Cross-sectional study*—Give the eligibility criteria, and the sources and methods of selection of participants	4
		(*b*) *Cohort study*—For matched studies, give matching criteria and number of exposed and unexposed<br>*Case-control study*—For matched studies, give matching criteria and the number of controls per case	
Variables	7	Clearly define all outcomes, exposures, predictors, potential confounders, and effect modifiers. Give diagnostic criteria, if applicable	5
Data sources/ measurement	8*	For each variable of interest, give sources of data and details of methods of assessment (measurement). Describe comparability of assessment methods if there is more than one group	*5*
Bias	9	Describe any efforts to address potential sources of bias	
Study size	10	Explain how the study size was arrived at	5
Quantitative variables	11	Explain how quantitative variables were handled in the analyses. If applicable, describe which groupings were chosen and why	5
Statistical methods	12	(*a*) Describe all statistical methods, including those used to control for confounding	5
		(*b*) Describe any methods used to examine subgroups and interactions	
		(*c*) Explain how missing data were addressed	
		(*d*) *Cohort study*—If applicable, explain how loss to follow-up was addressed<br>*Case-control study*—If applicable, explain how matching of cases and controls was addressed<br>*Cross-sectional study*—If applicable, describe analytical methods taking account of sampling strategy	

For peer review only - http://bmjopen.bmj.com/site/about/guidelines.xhtml

(*e*) Describe any sensitivity analyses

Continued on next page

For peer review only

Results			Page in the manuscript
Participants	13*	(a) Report numbers of individuals at each stage of study—eg numbers potentially eligible, examined for eligibility, confirmed eligible, included in the study, completing follow-up, and analysed	6
		(b) Give reasons for non-participation at each stage	
		(c) Consider use of a flow diagram	
Descriptive data	14*	(a) Give characteristics of study participants (eg demographic, clinical, social) and information on exposures and potential confounders	6
		(b) Indicate number of participants with missing data for each variable of interest	
		(c) *Cohort study*—Summarise follow-up time (eg, average and total amount)	
Outcome data	15*	*Cohort study*—Report numbers of outcome events or summary measures over time	
		Case-control study—Report numbers in each exposure category, or summary measures of exposure	
		Cross-sectional study—Report numbers of outcome events or summary measures	*6*
Main results	16	(*a*) Give unadjusted estimates and, if applicable, confounder-adjusted estimates and their precision (eg, 95% confidence interval). Make clear which confounders were adjusted for and why they were included	6
		(*b*) Report category boundaries when continuous variables were categorized	
		(*c*) If relevant, consider translating estimates of relative risk into absolute risk for a meaningful time period	
Other analyses	17	Report other analyses done—eg analyses of subgroups and interactions, and sensitivity analyses	
**Discussion**			
Key results	18	Summarise key results with reference to study objectives	7-9
Limitations	19	Discuss limitations of the study, taking into account sources of potential bias or imprecision. Discuss both direction and magnitude of any potential bias	9
Interpretation	20	Give a cautious overall interpretation of results considering objectives, limitations, multiplicity of analyses, results from similar studies, and other relevant evidence	7-9
Generalisability	21	Discuss the generalisability (external validity) of the study results	7-10
**Other information**			
Funding	22	Give the source of funding and the role of the funders for the present study and, if applicable, for the original study on which the present article is based	11

*Give information separately for cases and controls in case-control studies and, if applicable, for exposed and unexposed groups in cohort and cross-sectional studies.

For peer review only

Note: An Explanation and Elaboration article discusses each checklist item and gives methodological background and published examples of transparent reporting. The STROBE checklist is best used in conjunction with this article (freely available on the Web sites of PLoS Medicine at http://www.plosmedicine.org/, Annals of Internal Medicine at http://www.annals.org/, and Epidemiology at http://www.epidem.com/). Information on the STROBE Initiative is available at www.strobe-statement.org.

For peer review only

For peer review only - http://bmjopen.bmj.com/site/about/guidelines.xhtml
